# Promoter R-Loops Recruit U2AF1 to Modulate Its Phase Separation and RNA Splicing

**DOI:** 10.1021/jacs.3c08204

**Published:** 2023-09-21

**Authors:** Xiaomei He, Jun Yuan, Zi Gao, Yinsheng Wang

**Affiliations:** † Department of Chemistry, University of California Riverside, Riverside, California 92521-0403, United States; ‡ Environmental Toxicology Graduate Program, University of California Riverside, Riverside, California 92521-0403, United States

## Abstract

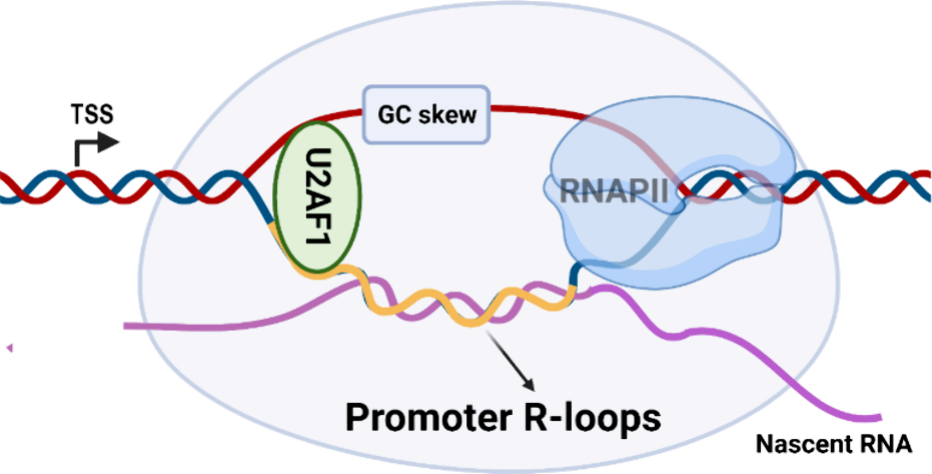

R-loops and guanine quadruplexes (G4s) are secondary structures of nucleic acids that are ubiquitously present in cells and are enriched in promoter regions of genes. By employing a bioinformatic approach based on overlap analysis of transcription factor chromatin immunoprecipitation sequencing (ChIP-seq) data sets, we found that many splicing factors, including U2AF1 whose recognition of the 3′ splicing site is crucial for pre-mRNA splicing, exhibit pronounced enrichment at endogenous R-loop- and DNA G4-structure loci in promoter regions of human genes. We also revealed that U2AF1 binds directly to R-loops and DNA G4 structures at a low-nM binding affinity. Additionally, we showed the ability of U2AF1 to undergo phase separation, which could be stimulated by binding with R-loops, but not duplex DNA, RNA/DNA hybrid, DNA G4, or single-stranded RNA. We also demonstrated that U2AF1 binds to promoter R-loops in human cells, and this binding competes with U2AF1’s interaction with 3′ splicing site and leads to augmented distribution of RNA polymerase II (RNAPII) to promoters over gene bodies, thereby modulating cotranscriptional pre-mRNA splicing. Together, we uncovered a group of candidate proteins that can bind to both R-loops and DNA G4s, revealed the direct and strong interactions of U2AF1 with these nucleic acid structures, and established a biochemical rationale for U2AF1’s occupancy in gene promoters. We also unveiled that interaction with R-loops promotes U2AF1’s phase separation, and our work suggests that U2AF1 modulates pre-mRNA splicing by regulating RNAPII’s partition in transcription initiation versus elongation.

## Introduction

R-loops are three-stranded nucleic acid structures consisting of an RNA/DNA hybrid and a displaced single-stranded DNA (ssDNA).^1^ Primarily formed during transcription, R-loops have gained substantial attention owing to their important roles in gene regulation, DNA damage repair, and genome stability maintenance.^1^ In recent years, R-loop structures in mammalian cells have been profiled at the genome-wide scale with several high-throughput sequencing methods, for example, DNA/RNA immunoprecipitation sequencing (DRIP-seq) and DNA-RNA in vitro enrichment coupled to sequencing (DRIVE-seq),^2^ R-chromatin immunoprecipitation sequencing (R-ChIP-seq),^3^ MapR,^4^ R-loop CUT&Tag,^5^ and DRIPc-seq^6^ These studies revealed that a GC skew, i.e., asymmetric enrichment of guanines over cytosines, in a nontemplate strand strongly favors R-loop formation, which may be attributed to the formation of DNA secondary structures, for example, guanine quadruplexes (G4s), in the displaced nontemplate strand. Such secondary structures augment R-loop stability through preventing the reannealing of double-stranded DNA and/or the access of R-loops by helicases.^1,7^

DNA G4s, four-stranded structures formed in G-rich regions of the genome, occur primarily in promoter regions of actively transcribed genes in human cells.^8,9^ R-loops and G4s are formed at loci displaying similar genomic feature (i.e., GC skew), and genome-wide profiling of R-loops and G4s in K562 cells has revealed considerable coenrichment of these structures in chromatin.^3,10,11^ Moreover, the coenrichment occurs mainly in promoter regions,^3,10,11^ indicating a functional interplay of R-loops and G4s in gene regulation.

By employing an in vitro transcription assay, Belotserkovskii et al.^12^ revealed that G-rich sequences in the nontemplate strand elicit transcription blockage through the formation of the R-loop structure, and the blockage was much more pronounced if the G-rich sequences are proximal to the promoter region. A more recent study demonstrated that R-loop induces G4 formation in the nontemplate strand, which in turn stabilizes the R-loop and promotes transcription,^13^ and another study also revealed a positive feedback mechanism between G4 and R-loop formation.^14^ Moreover, G4 ligands can induce DNA damage and give rise to genomic instability in human cancer cells in an R-loop-dependent manner.^15^ Together, these previous studies suggest that the R-loop and G4 structures are highly correlated in the human genome, and the regulatory roles of R-loops and G4s in gene expression may be functionally coupled.

On the grounds that the regulatory functions of R-loops and G4s in cells are exerted through cellular proteins, many studies have been conducted for the identification and functional characterizations of R-loop- or G4-recognition proteins. Using affinity purification with the S9.6 antibody, which recognizes RNA/DNA hybrids in a sequence-independent manner,^16,17^ coupled with mass spectrometry (MS)-based quantitative proteomics, Cristini et al.^18^ investigated the RNA/DNA hybrid interactome in HeLa cells. They identified DHX9 as an R-loop-binding protein, and this binding modulates transcription termination and DNA damage response.^18^ More recently, Mosler et al.^19^ developed an RNA–DNA proximity proteomics method by fusing ascorbate peroxidase 2 (APEX2) with the DNA/RNA hybrid-binding domain of RNase H1 and identified DDX41 as an R-loop helicase. Furthermore, Williams et al.^20−23^ employed a quantitative proteomics method relying on affinity purification with three biotinylated G4 DNA probes and the corresponding ssDNA probes and identified multiple G4-binding proteins, including SLIRP, YY1, VEZF1, and so on. There is no report, however, on the identification of cellular proteins that can interact with both R-loop and G4 structures. We reason that such a study will provide important knowledge for understanding the functional interplay between R-loops and G4s in gene regulation.

In this study, we interrogated publicly available ChIP-seq data and uncovered a group of candidate proteins that can bind to both R-loops and DNA G4s. We also revealed that one of these proteins, U2AF1, can interact directly with R-loops at low-nM binding affinity, where the interaction enhances U2AF1’s ability to undergo liquid–liquid phase separation and modulates U2AF1’s function in pre-mRNA splicing.

## Results

### Bioinformatic Discovery of R-Loop- and G4-Binding Proteins

**Figure 1:**
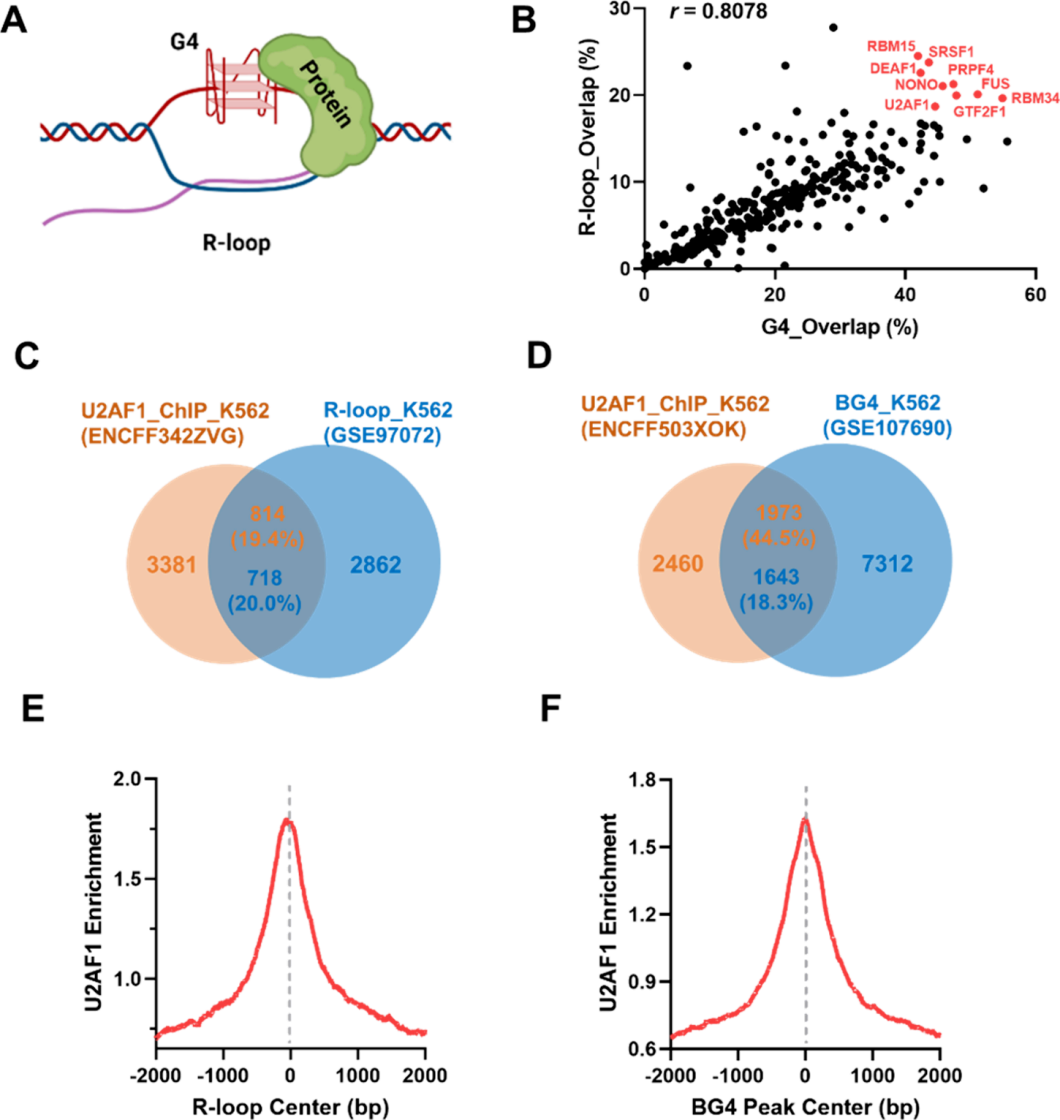
Bioinformatic discovery of candidate R-loop- and DNA G4-binding proteins. (A) A schematic diagram illustrating the co-occurrence of R-loop and G4 structures and their recognition by cellular proteins. (B) A scatter plot illustrating the percentages of overlap of transcription factor binding sites with G4 and R-loop loci in chromatin. (C, D) Venn diagrams illustrating the overlapping peak numbers and the percentages of overlap of the U2AF1 ChIP-seq data set with the R-ChIP-seq data set (C) and BG4-ChIP-seq data set (D) in K562 cells. (E, F) Aggregation plots depicting the colocalization of U2AF1 binding sites with R-loop (E) and G4 structure (F) loci in the human genome.

We recently developed a bioinformatic approach for the identification of RNA G4 (rG4)-binding proteins based on an overlapping analysis, at the entire transcriptome scale, of rG4 structure loci and binding sites of RNA-binding proteins (RBPs) with the use of publicly available rG4-seq data set and eCLIP-seq data sets, respectively.^24^ Similarly, by comparing DNA G4 loci with binding sites of various chromatin-associated proteins and histone epigenetic marks derived from ChIP-seq data in ENCODE, Spiegel et al.^25^ revealed that endogenous G4s constitute binding hubs for transcription factors to modulate transcription. We reason that a similar overlapping analysis of the binding sites of proteins with R-loop and G4 structure loci in the human genome may allow for the identification of proteins that can bind to both R-loop and G4 structures. Thus, we developed a bioinformatic method for the discovery of candidate R-loop- and G4-binding proteins based on publicly available R-ChIP-seq,^3^ DNA G4 ChIP-seq,^10^ and over 600 transcription factor ChIP-seq data sets^26^ generated from human cells (Figure 1A).

Such an analysis yielded overlapped percentages for 322 proteins with R-loop and G4 structure sites (Tables S1 and S2). A scatter plot revealed that the percentages of overlapped binding sites of different proteins with G4 structure and R-loop loci are strongly correlated (*r* = 0.8078, Figure 1B), suggesting that a large number of proteins are coenriched at R-loop and G4 structure sites in chromatin. Several of these proteins (highlighted in Figure 1B) exhibited very high percentages of overlap with both the G4 structures and R-loop sites. Among these proteins, SRSF1, NONO, and FUS are known DNA and RNA G4-binding proteins,^25,27−29^ and SRSF1, PRPF4, NONO, and GTF2F1 have been previously documented as candidate R-loop-binding proteins.^18,19^ Thus, our results revealed cellular proteins known to recognize R-loop and G4 structures, underscoring the feasibility of the method in discovering novel R-loop- and G4-binding proteins.

### U2AF1 Is a R-Loop-Binding Protein

We found that several highly ranked candidate R-loop- and G4-binding proteins, that is, U2AF1, SRSF1, NONO, RBM15, RBM34, and PRPF4, are known to be involved in mRNA splicing, indicating that splicing factors may exert their functions partly through binding with R-loops and DNA G4s. In this vein, Chen et al.^30^ recently showed that myelodysplastic syndrome-linked Q157P mutation in U2AF1 could give rise to augmented R-loops in both promoter and nonpromoter regions in human cells and elicit elevated occupancy of RNA polymerase II (RNAPII) at the transcription start site relative to the gene body. The underlying biochemical mechanism, however, remained elusive. Hence, we decided to focus our subsequent study on U2AF1.

We first analyzed in detail the overlaps of the U2AF1 ChIP-seq data set with BG4-ChIP-seq and R-ChIP-seq data sets in K562 cells (Figure 1C,D). We found that 50% of U2AF1 ChIP-seq peaks are enriched in promoter regions (Figure S1A), and an even higher percentage (∼70%) of promoter localization was observed for those U2AF1 peaks that are colocalized with both R-loop and G4 peaks (Figure S1B–D). Aggregation plots revealed that the U2AF1 ChIP-seq signal was highly enriched at the R-loop and G4 peak centers (Figure 1E–F). A similar analysis of BG4 and U2AF1 ChIP-seq data sets generated from HepG2 cells again revealed a high percentage of co-occupancy of U2AF1 with G4 loci. In particular, 46.6% of U2AF1 peaks are localized in promoters, and an even greater percentage (∼60.9%) of U2AF1/G4 co-occupancy sites reside in promoters (Figure S2). Thus, our bioinformatic analysis suggests that the high level of U2AF1 occupancy in gene promoters is attributed to its interactions with R-loop and G4 structures in the promoter regions.

**Figure 2:**
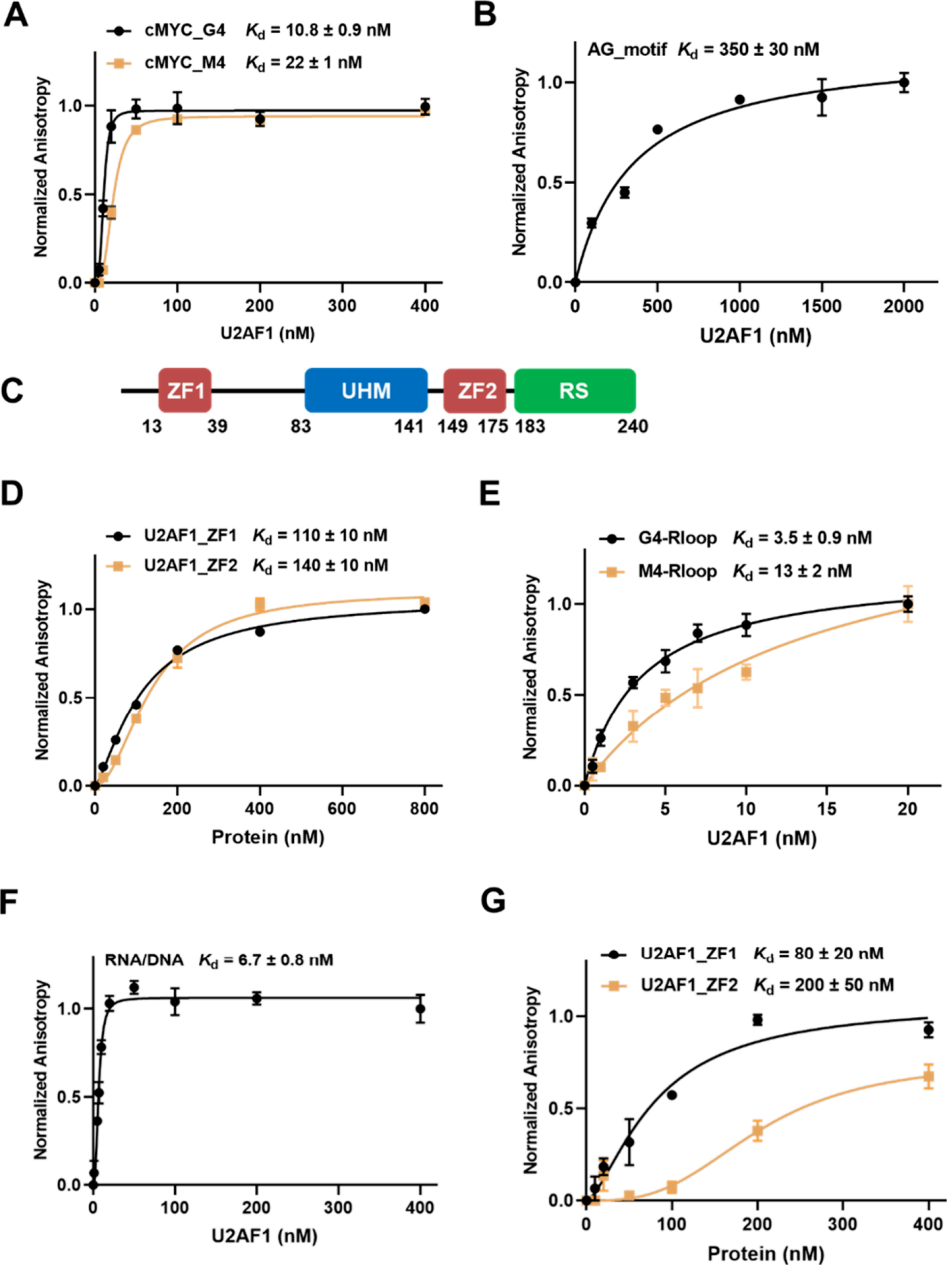
U2AF1 binds directly to G4 DNA and R-loop, and both ZF domains of U2AF1 are involved in U2AF1–nucleic acid interactions. (A,B, E,F, H,I) Fluorescence anisotropy results showing the binding affinities of full-length U2AF1 toward *cMYC* G4 and M4 (A), AG motif-harboring ssRNA (B), G4- and M4-R-loops (E), and RNA/DNA hybrid (F). (C) A schematic diagram depicting the domain structure of U2AF1 protein. (D,G) Fluorescence anisotropy results showing the binding affinities of two truncated mutants of U2AF1 toward cMYC G4 (D) and RNA/DNA hybrid (G). Error bars represent SEM (*n* = 3).

We recognized that the colocalization of U2AF1 with these structures may arise from indirect interactions through other proteins. Thus, we purified the recombinant U2AF1 protein (Figure S3A) and examined its abilities in binding directly with different nucleic acid structures. We first employed previously reported fluorescently labeled DNA G4 probes,^31^ including two parallel G4 folding probes derived from the promoters of *cMYC* and *cKIT* genes, an antiparallel (2KF8), and a hybrid-type G4 (hTel) folding probe derived from the human telomere, as well as their corresponding mutant probes (M4) incompetent in folding into G4 structures (Table S3), to assess U2AF1-G4 interactions. Fluorescence anisotropy results showed that U2AF1 binds strongly toward G4s, with the *K*_d_ values being 10.8 ± 0.9, 15 ± 2, 9 ± 1, and 50 ± 6 nM for G4s derived from *cMYC, cKIT*, 2KF8, and hTel, respectively (Figure 2A and Figure S3B–D). We also observed interactions between U2AF1 and M4s, with similar or slightly poorer binding affinities (*K*_d_ = 22 ± 1, 19 ± 4, 10 ± 2, and 60 ± 6 nM, respectively, Figure 2A and Figure S3B–D). These results demonstrated that U2AF1 binds directly with DNA G4s at low-nM binding affinity, where G4s in parallel and antiparallel folding topologies display similar binding affinities toward U2AF1.

To confirm the findings made from fluorescence anisotropy measurements, we employed the electrophoretic mobility shift assay (EMSA) to determine the binding affinities of U2AF1 toward *cMYC* and *cKIT* DNA probes. The results showed that the *K*_d_ values obtained from EMSA were very similar to those obtained from fluorescence anisotropy experiments, indicating strong binding affinities of U2AF1 toward G4 and M4 probes, with a moderate preference in binding toward G4 over M4 probes (Figure S4A–D). Since both G4 and M4 probes consist of G-rich sequences, we next investigated whether G4 folding promotes the binding of the G4 probe to U2AF1. To this end, we conducted EMSA experiments using the *cMYC* G4 probe annealed in a Li^+^ buffer, which is known to prevent G4 folding. Our results showed that the *K*_d_ value of U2AF1 toward *cMYC* G4 in the Li^+^ buffer was 8 times higher than that in the K^+^ buffer (Figure S4E), suggesting the role of G4 folding in promoting the binding of U2AF1 toward the *cMYC* G4 probe.

We also examined whether binding to U2AF1 alters the folding of the G4 structure. The circular dichroism (CD) spectrum for the *cMYC* G4 DNA probe in the U2AF1-DNA complex was very similar to that of the free G4 DNA probe (Figure S5A), substantiating that the DNA maintains G4 folding when it binds with U2AF1.

Previous biochemical and structural studies revealed that U2AF1 recognizes the AG dinucleotide in its RNA motif sequence (5′-UUAGGU-3′) at the 3′ splicing site, where the *K*_d_ value of U2AF1 binding toward the AG motif was 0.47 μM based on isothermal titration calorimetry (ITC) measurement.^32^ For comparison, we also measured the binding affinity of U2AF1 for a fluorescently labeled AG motif using fluorescence anisotropy. Our results showed that the *K*_d_ value for U2AF1’s binding toward the AG motif obtained from anisotropy measurement (350 nM) was very similar to the previously reported ITC result (Figure 2B). Thus, U2AF1 exhibited much stronger interactions with G4 and M4 DNA than with AG-motif-harboring RNA.

Human U2AF1 protein contains four domains, a central U2AF homology motif (UHM) flanked with two CCCH-type zinc finger domains (i.e., ZF1 and ZF2) and a C-terminal RS domain (Figure 2C), where the ZF and UHM domains are highly conserved in eukaryotes, and the two ZFs assume dominant roles in U2AF1-RNA interaction.^33^ To investigate how U2AF1 recognizes DNA G4, we have purified truncated variants of the U2AF1 protein containing only ZF1 or ZF2 (Figure S3A) and measured their binding affinities toward *cMYC* G4. Our results showed that both ZFs displayed direct interactions with G4, albeit with binding affinities being over 10-fold lower than that of full-length U2AF1 (Figure 2D). These results are consistent with the previous finding that the two ZFs in U2AF1 can each form a binding surface and bind cooperatively to target RNA sequence.^33^ Therefore, the two ZFs of U2AF1 contribute to the protein’s direct interactions with both the RNA and G4 DNA.

We next examined the binding of U2AF1 to DNA G4 and M4 in R-loop structures. To this end, we designed bubble-structured G4- and M4-R-loop probes with a displaced ssDNA harboring *cMYC* G4 and M4 sequences, respectively (Table S3). The successful formation of R-loop structures was confirmed by native PAGE analysis and CD spectroscopy (Figures S3C and S5B). In this vein, the CD spectra of G4-R-loop and M4-R-loop were similar, displaying two positive peaks at around 185 and 275 nm, which are in agreement with previous observations.^34^ The results from both fluorescence anisotropy and EMSA revealed that the full-length U2AF1 protein interacts directly with G4- and M4-R-loops, displaying very strong binding affinities. Specifically, the *K*_d_ value of the G4-R-loop probe was slightly lower than that of the M4-R-loop probe (Figure 2E and Figure S6). The higher binding affinity of U2AF1 toward the G4-R-loop than the M4-R-loop indicates that U2AF1’s ability in recognizing *cMYC* G4 is preserved when it is situated on the displaced ssDNA of the R-loop structure.

Our EMSA results also showed that the G4-R-loop probe, but not the M4-R-loop probe, displays some slow migrating species (Figure S6). Previous studies revealed that G4 structures in the R-loop can form intramolecularly on the displaced ssDNA or intermolecularly between the RNA strand and the displaced ssDNA.^35,36^ Additionally, Liano et al.^37^ showed recently that CSB protein selectively binds to and unwinds intermolecular G4s but not intramolecular G4s. On the basis of these previous findings, we reason that the slow migrating species observed in the EMSA gel might be attributed to the formation of intermolecular G4s. U2AF1, nonetheless, does not exhibit apparent preference in binding toward intermolecular over intramolecular G4-R-loop (Figure S6).

Since the R-loop contains a displaced ssDNA and an RNA/DNA hybrid, we next asked whether U2AF1 interacts with the RNA/DNA hybrid. To this end, we employed an RNA/DNA hybrid probe with RNA sequence derived from the aforementioned R-loops for the binding experiments (Table S3). We found that the binding affinity of U2AF1 toward the RNA/DNA hybrid (*K*_d_ = 6.7 nM) was lower than that toward the G4 R-loop (3.5 nM) but higher than that toward the M4 R-loop (13 nM, Figure 2F). We also found that ZF1 and ZF2 of U2AF1 exhibit weak interactions with the RNA/DNA hybrid (Figure 2G). Together, these results suggest that RNA/DNA hybrid and the displaced ssDNA cooperatively contribute to U2AF1’s interaction with R-loops.

We next investigated whether U2AF1 interacts with double-stranded DNA (dsDNA) harboring the same sequence as the RNA/DNA hybrid (Table S3). The results showed that U2AF1 is capable of interacting with dsDNA, though the *K*_d_ value for binding toward dsDNA was much higher than that toward the RNA/DNA hybrid (Figure S3F). Our above results together revealed that U2AF1 binds directly to R-loops and the RNA/DNA hybrid at superior binding affinities than toward dsDNA.

### R-Loop Binding Promotes the Phase Separation of U2AF1

**Figure 3:**
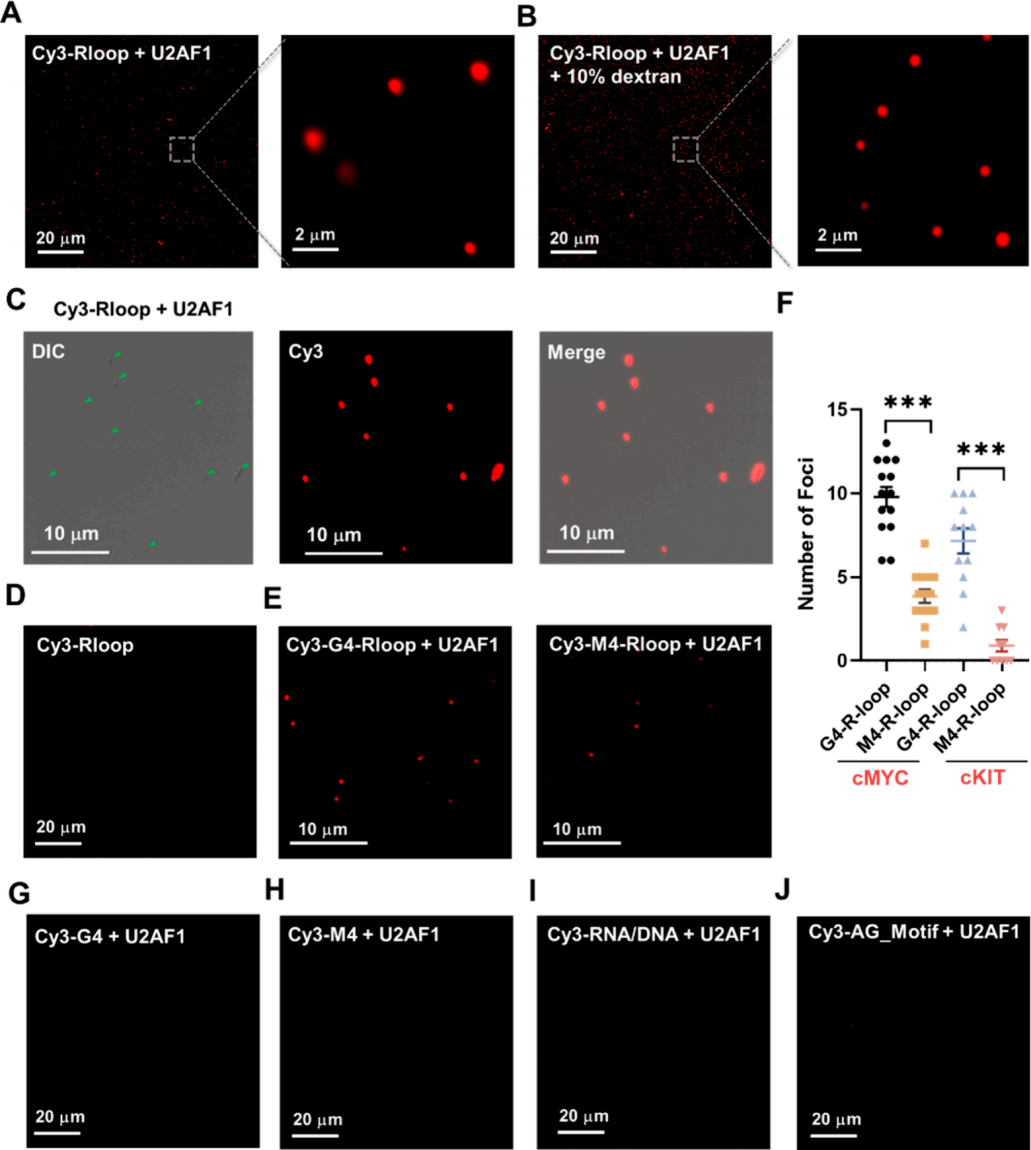
The U2AF1-R-loop complex undergoes phase separation in vitro. (A,B) Fluorescence images showing the phase separation of U2AF1-R-loop in the absence (A) or presence (B) of 10% dextran. (C) DIC and fluorescence images of the U2AF1-R-loop mixture. (D) The R-loop itself does not undergo phase separation. (E) Flurescence images showing the phase separation of U2AF1-R-loop induced by G4 or M4-R-loop. (F) Quantitative results from data in panel (E) for the number of foci in G4- or M4-R-loop-induced phase separtion. The *p* values were calculated by using unpaired, two-tailed Student’s *t*-test. ***p < 0.001. (G–J) Fluorescence images illustrating the inabilities of DNA G4 (G), DNA M4 (H), RNA/DNA hybrid (I), or AG motif (J) in promoting the phase separation of the U2AF1 protein in vitro. The concentration of U2AF1 in these solutions was 500 nM. Green solid triangles indicate the locations of droplets.

During fluorescence anisotropy measurements for U2AF1-R-loop complexes, we observed unexpectedly negative anisotropy values when the concentration of U2AF1 exceeded 50 nM (Figure S7A). This observation prompted us to reason that binding with the R-loop may stimulate U2AF1 to undergo phase separation. To explore this possibility, we examined the mixture of fluorescently labeled R-loop and U2AF1 using confocal microscopy. Indeed, we observed phase separation when the molar ratio of U2AF1/R-loop reached 5:1, and the number of phase-separated foci increased with the rise of protein concentration (Figures 3A and Figure S7B), which is in keeping with our findings made from fluorescence anisotropy measurements. To mimic the crowded intracellular environment, we added 10% dextran, a crowding agent,^38,39^ to the mixture. The results showed that dextran greatly enhanced droplet formation in the U2AF1-R-loop mixture (Figure 3B).

We next investigated whether the U2AF1 protein and R-loop can undergo phase separation by imaging analysis using fluorescence and differential interference contrast (DIC) microscopy. DIC images revealed that U2AF1 itself exhibits weak ability in undergoing phase separation (Figure S8A), which is augmented by inclusion of 10% dextran or R-loop in the solution (Figure S8B and Figure 3B,C). Additionally, the DIC image of U2AF1-R-loop droplets overlapped completely with the foci in the corresponding fluorescence image, underscoring that the fluorescently labeled R-loop is recruited into U2AF1 protein droplets (Figure 3C). Furthermore, the phase separation of U2AF1 is significantly disrupted by 1,6-hexanediol (1,6-HD, Figure S8C,D), which inhibits hydrophobic protein–protein or protein–RNA interactions required for liquid droplet formation.^40^ This result showed that most U2AF1 droplets are in liquid-like condensates, and hydrophobic interactions contribute to liquid–liquid phase separation (LLPS) of U2AF1. No foci, however, could be observed for the R-loop itself (Figure 3D). Together, these observations revealed that U2AF1 can undergo phase separation in vitro, which is substantially enhanced by R-loops.

Given that U2AF1 exhibits preferential binding toward the G4- over M4-R-loop, we next assessed the effect of fluorescently labeled G4- and M4-R-loops on modulating phase separation of U2AF1. Fluorescence images showed that the inclusion of G4-R-loop results in a more pronounced enhancement in phase separation of U2AF1 than that of M4-R-loop (Figure 3E,F). This is further substantiated by in vitro results obtained from another pair of G4- and M4-R-loops derived from *cKIT* promoter (Figure S7C and Figure 3F). Since our above-mentioned results showed that U2AF1 binds to G4, M4, and RNA/DNA hybrids with very high affinities, we also examined the abilities of these nucleic acid structures in promoting U2AF1 droplet formation. Interestingly, the addition of G4, M4, RNA/DNA hybrid, or AG motif into U2AF1 protein solution failed to increase U2AF1’s tendency to undergo phase separation (Figure 3G–J). Thus, the ability of nucleic acids to elicit phase separation of U2AF1 depends on not only their binding affinities with U2AF1 but also their secondary structures. Among all of the nucleic acid structures examined, only R-loops stimulate the phase separation of U2AF1 protein, with G4-R-loops being more effective than the corresponding M4-R-loops.

**Figure 4:**
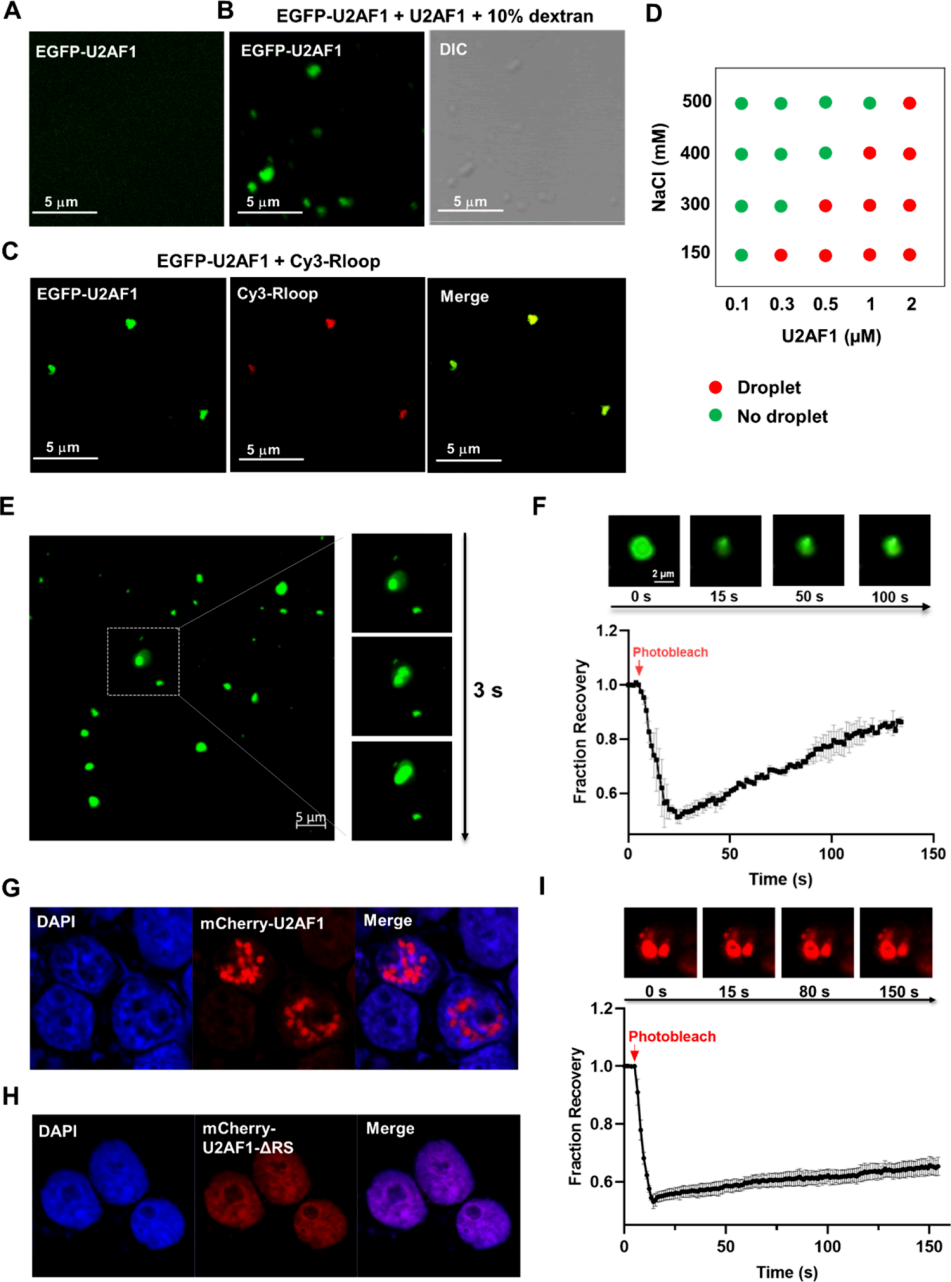
U2AF1 undergoes phase separation in vitro and in cells, which is modulated by its C-terminal RS domain. (A) EGFP-U2AF1 protein does not undergo phase separation in vitro. (B) EGFP-U2AF1 undergoes phase separation upon the addition of unlabeled U2AF1 and 10% dextran. (C) The colocalization of EGFP-U2AF1 and R-loop foci, showing that both components are recruited into phase-separated liquid droplets. The molar ratio of R-loop to EGFP-U2AF1 is 1:5. (D) The influences of protein and NaCl concentrations on the formation of phase-separated liquid droplets, where red and green circles indicate the presence and absence of phase-separated droplets, respectively. The experiments were conducted using a mixture of an equal amount of EGFP-U2AF1 and unlabeled U2AF1, supplemented with 10% dextran. (E) Fluorescence images of EGFP-U2AF1 (spiked with an equal amount of unlabeled U2AF1) revealed the rapid fusion of droplets (within several sec). (F) FRAP of EGFP-U2AF1/unlabeled U2AF1 at equal amount with 10% dextran. Error bar represents SD (*n* = 3). (G,H) mCherry-U2AF1 undergoes phase separation in cells (G), whereas mCherry-U2AF1-ΔRS fails to do so (H). (I) Cellular FRAP result showing the poor fluorescence recovery of U2AF1 foci after photobleaching in cells. Error bars represent SEM (*n* = 3).

To gain insights into the phase separation property of U2AF1, we purified recombinant EGFP-U2AF1 and conducted in vitro phase separation experiments (Figure S9A). Fluorescence images revealed that EGFP-U2AF1 solution is homogeneous, indicating that EGFP-U2AF1 itself was unable to undergo phase separation (Figure 4A), which is in agreement with the previous observation that EGFP tagging can increase the solubility of the fusion protein, thereby perturbing the phase separation dynamics of the tagged protein.^41^ By adding an equal amount of untagged U2AF1 and 10% dextran, we observed that EGFP foci and droplets are overlapped with each other (Figure 4B), indicating the condensation of the U2AF1 protein into droplets. Moreover, the R-loop can induce the phase separation of EGFP-U2AF1, where fluorescence microscopy analysis confirmed the enrichment of the R-loop and EGFP-U2AF1 in the droplets (Figure 4C).

To gain additional insights into the intermolecular interactions underlying the phase separation of U2AF1, we mapped the phase diagram of U2AF1 in the presence of dextran, along with increasing concentrations of the protein or NaCl. We observed that increasing NaCl concentration confers a higher threshold protein concentration required for phase separation (Figure 4D), indicating that electrostatic interactions also contribute to phase separation of U2AF1. Along with the result obtained from the 1,6-HD experiment (Figure S8C,D), our observations suggest that both hydrophobic and electrostatic interactions facilitate LLPS of U2AF1.

We also monitored the dynamic properties of U2AF1 droplets by employing droplet fusion and fluorescence recovery after photobleaching (FRAP) assays. Our results showed that U2AF1 droplets grow into larger droplets within seconds (Figure 4E), which is in agreement with the liquid property of the protein condensates. However, when the size reaches ∼1 μm, many droplets aggregate together and become irregularly shaped over time, indicating a liquid- to gel-like transition. A similar finding was made from FRAP assay, where we observed that the gel-like property of droplets led to a poor recovery in fluorescence after photobleaching (Figure 4F). These in vitro phase transitions are reminiscent of previous observations made for two prion-like RBPs, that is, TDP-43 and FUS.^38,39^ Previous studies also documented that low RNA/protein ratios promote phase separation of prion-like RBPs, whereas high ratios impede droplet formation in vitro.^38^ Thus, we also examined whether U2AF1 behaves in a similar way by conducting in vitro phase separation experiments using a fixed concentration of U2AF1 and increasing concentrations of R-loop. Our results showed that the addition of the R-loop facilitated LLPS of U2AF1 at low R-loop/protein molar ratios (less than 0.2). Marked decreases in both the number and size of droplets were, however, observed when the molar ratios exceed 0.3 (Figure S9B). These results indicate that the material properties of U2AF1 droplets resemble those of some prion-like RBPs, and the R-loop can modulate the phase separation behavior of U2AF1 in vitro.

We next examined the phase separation of U2AF1 in cells. To this end, we ectopically expressed mCherry-U2AF1 in HEK293T cells. Fluorescence micrographs revealed that mCherry-U2AF1 undergoes phase separation in cells, where the foci are located in the nuclei (Figure 4G). We also explored which domain of U2AF1 mediates the phase separation. Based on the IUPred2 prediction,^42^ the C-terminal RS domain of U2AF1 is an intrinsically disordered region (IDR, Figure S9C), which plays crucial roles in regulating LLPS of many proteins.^38,43,44^ Our results showed that mCherry-U2AF1-ΔRS, a truncated variant of U2AF1 with the RS domain deleted, failed to assemble into protein condensates in cells (Figure 4H), suggesting that the RS domain is indispensable for the LLPS of U2AF1. In addition, deletion of the ZF1 (mCherry-U2AF1-ΔZF1) or ZF2 (mCherry-U2AF1-ΔZF2) domain in U2AF1 led to diminished formation of U2AF1 foci (Figure S10A,B), indicating that ZF domains also contribute to LLPS of U2AF1 in cells.

We next conducted live-cell imaging to examine the material properties of the U2AF1 droplets. The results showed that 1,6-HD perturbed the assembly of U2AF1 droplets in cells in minutes, although the foci intensity was not substantially diminished (Figure S10C). Moreover, our FRAP experiment showed slow and incomplete recovery of the fluorescence intensity of U2AF1 droplets after photobleaching (Figure 4I). These findings suggest that U2AF1 droplets undergo a liquid-to-gel transition in cells.

### U2AF1-R-Loop Interaction Interferes with U2AF1’s Recognition of the 3′ Splicing Site

**Figure 5:**
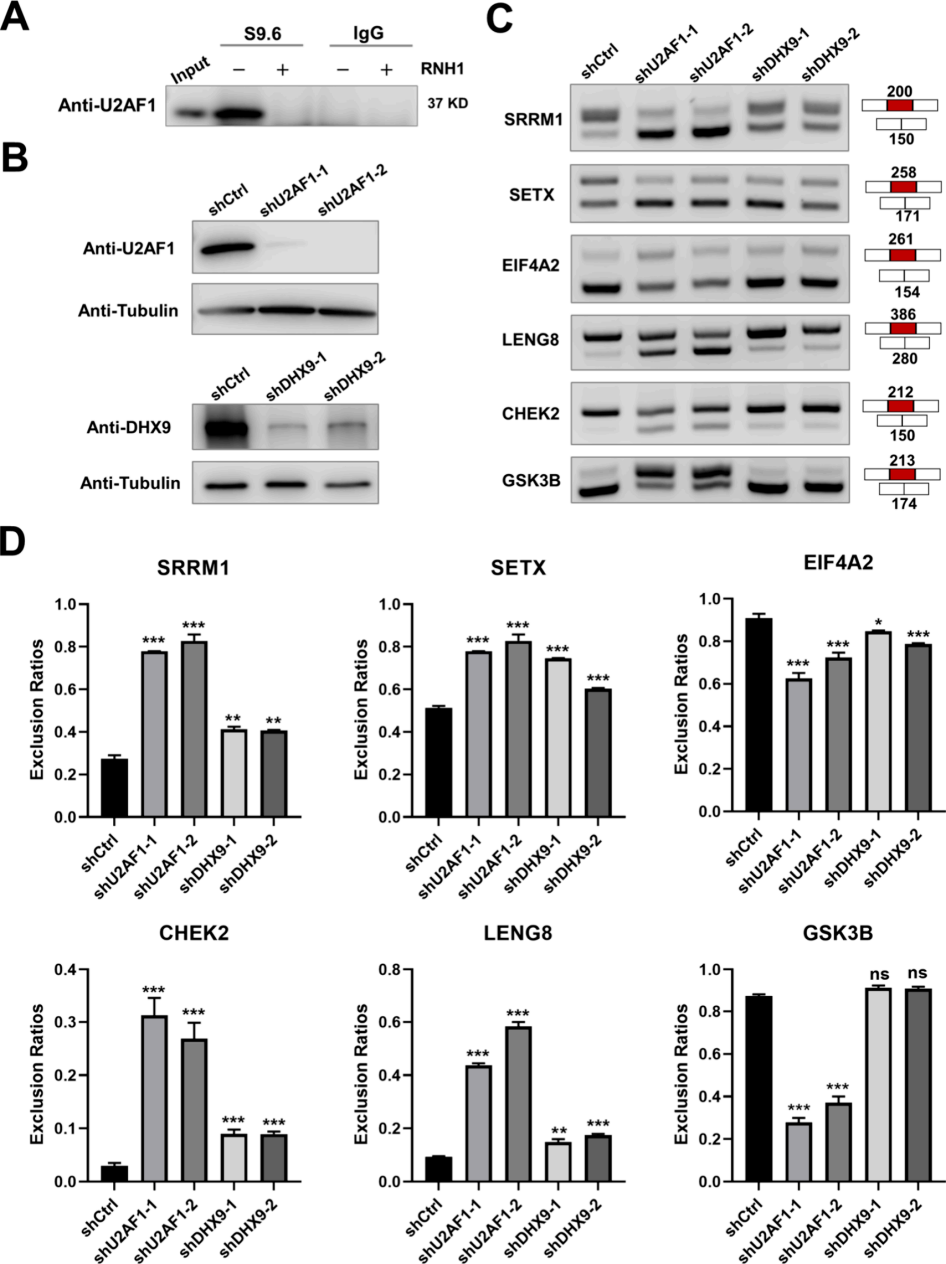
Genetic depletions of U2AF1 and DHX9 in cells exert similar effects on pre-mRNA splicing of R-loop-containing genes. (A) Immunoprecipitation by the S9.6 antibody revealed U2AF1-R-loop interactions in cells, which are disrupted by RNase H1 treatment. (B) Western blot confirming the depletion of U2AF1 and DHX9 in HEK293T cells. (C) RT-PCR analysis showing alternations in splicing of potential R-loop-containing genes upon genetic depletion of U2AF1 or DHX9 in HEK293T cells. (D) Quantification results of panel (C). Exclusion ratio = Exon exclusion/(Exon inclusion + Exon exclusion). Error bars represent SEM (*n* = 3). The *p* values were calculated by using unpaired, two-tailed Student’s *t*-test. * 0.01 ≤ *p* < 0.05; **0.001 ≤ *p* < 0.01; ***p < 0.001.

We next investigated U2AF1-R-loop interactions in cells by using immunoprecipitation with an S9.6 antibody, which specifically recognizes RNA/DNA hybrids at a subnanomolar binding affinity.^16^ The results showed that U2AF1 can be immunoprecipitated by the S9.6 antibody but not control IgG, and this precipitation was abolished after RNase H1 treatment (Figure 5A), suggesting that U2AF1 interacts with R-loops in cells.

The U2AF complex is known to define the 3′ splicing site in pre-mRNA splicing, and the recognition of the 3′ splicing site by U2AF1 is crucial for accurate splicing.^33,45^ We next explored the impact of the U2AF1-R-loop interaction on RNA splicing. To this end, we knocked down U2AF1 in HEK293T cells by using shRNA and confirmed the successful depletion of U2AF1 by Western blot (Figure 5B). Based on R-ChIP-seq and RNA-seq data sets,^3,45^ we chose six genes harboring potential R-loop structures in their promoter regions and exhibiting U2AF1-dependent splicing for reverse transcription-PCR (RT-PCR) analyses. We first checked the existence of R-loops in the promoter regions of these genes using R-chromatin immunoprecipitation quantitative real-time PCR (ChIP-qPCR) experiments, and the results showed that five out of the six genes contain R-loop structures in their promoter regions (Figure S11A). Moreover, RT-PCR results revealed that the changes in alternative splicing induced by U2AF1 depletion in HEK293T cells are consistent with the previous findings made for U2AF1-depleted HeLa cells (Figure 5C,D).^45^

DHX9 is a crucial helicase involved in R-loop resolution, and genetic depletion of DHX9 was found to increase the level of promoter R-loops in the human genome.^46^ Thus, we monitored the alternative splicing of these genes in DHX9-depleted HEK293T cells to examine how R-loops regulate alternative splicing. Our results showed that genetic depletion of DHX9 in HEK293T cells led to altered splicing similar to the knockdown of U2AF1 for the aforementioned five R-loop-harboring genes but not for the one without R-loop in its promoter region (Figure 5C,D).

**Figure 6:**
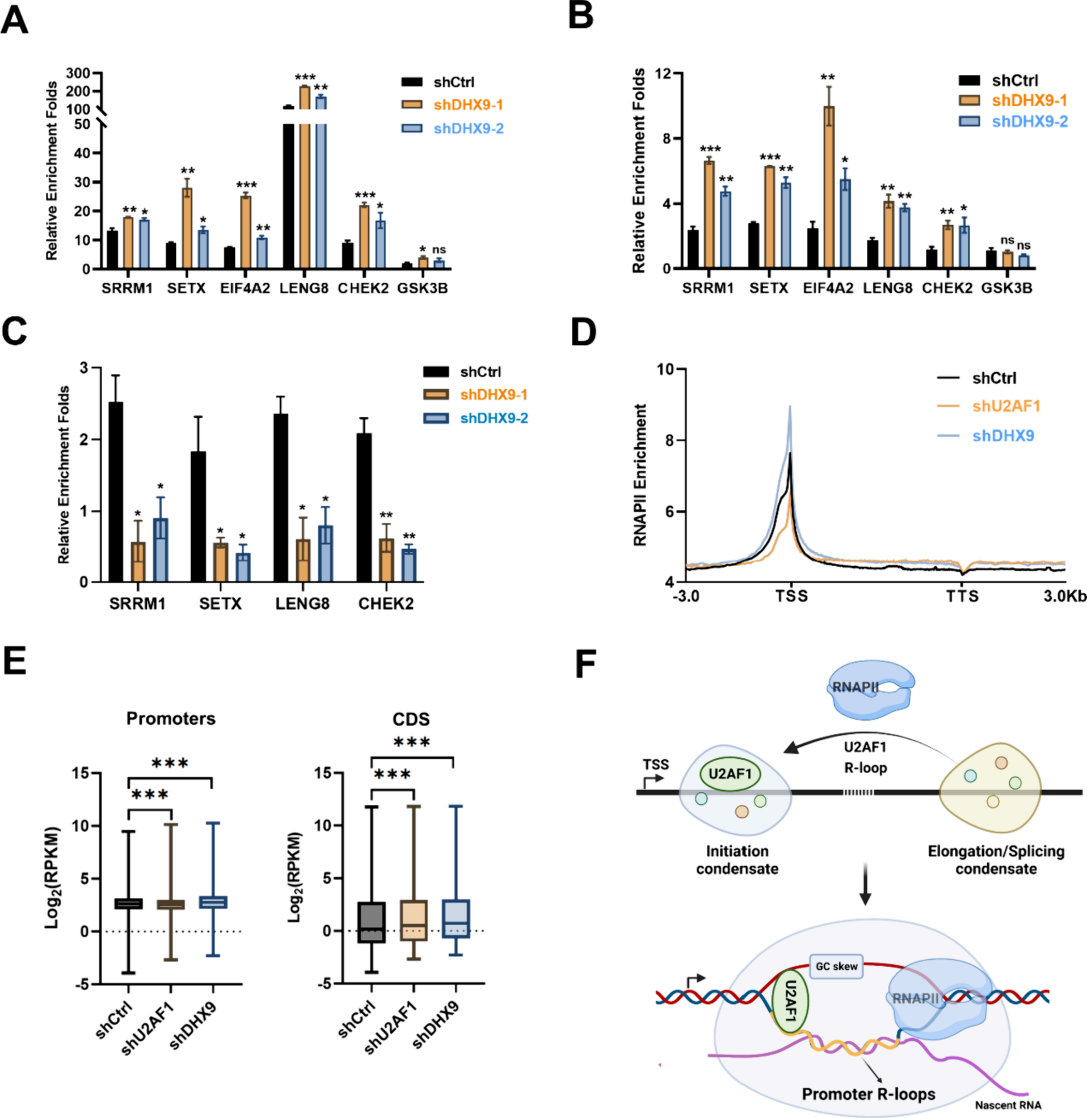
U2AF1-R-loop interaction regulates cotranscriptional splicing. (A) R-ChIP-qPCR results showing the augmented R-loop levels in promoter regions of four genes upon DHX9 depletion (A). (B) U2AF1 ChIP-qPCR data revealing that U2AF1’s occupancy at gene promoters is elevated in DHX9-depleted cells. (C) CLIP-qPCR results showing diminished enrichment of U2AF1 at the 3′ splicing site in DHX9-depleted cells. (D,E) Metagene profile (D) and ChIP-seq density analysis (E) revealing the effects of U2AF1 and DHX9 on the distribution of RNA RNAPII in the human genome. Error bars represent SEM (*n* = 3). The *p* values were calculated by using unpaired, two-tailed Student’s *t*-test. *0.01 ≤ *p* < 0.05; **0.001 ≤ *p* < 0.01; ***p < 0.001. (F) A model depicting the role of R-loops in modulating cotranscriptional splicing, in which R-loops recruit splicing factors (e.g., U2AF1) to the promoter regions of actively transcribed genes for cotranscriptional spliceosome assembly through LLPS. Additionally, U2AF1-R-loop interactions promote the partition of RNAPII into transcriptional initiation phase-separated condensates. Panel (F) was created with BioRender.com.

We reasoned that increased level of R-loops in DHX9-depleted cells may sequester U2AF1 at R-loop loci and impede U2AF1’s ability to interact with the 3′ splicing site. To test this, we first examined R-loop levels and U2AF1 occupancy in promoter R-loops in HEK293T and the isogenic DHX9-depleted cells using ChIP-qPCR experiments. Our results showed that DHX9 depletion led to significant elevations in both R-loop levels and U2AF1 occupancies in promoter regions of these genes with promoter R-loops but not the one without (Figure 6A,B). We also monitored the levels of U2AF1 at the 3′ splicing site by employing UV cross-linking, followed by immunoprecipitation and quantitative PCR (CLIP-qPCR) analysis. Our results revealed substantially diminished occupancy of U2AF1 at the 3′ splicing site upon genetic depletion of DHX9 (Figure 6C). Together, these results suggest that U2AF1’s interaction with promoter R-loops competes with its binding at the 3′ splicing site, thereby modulating RNA splicing. We, nevertheless, cannot exclude the possibility that the U2AF1-R-loop interaction may also alter RNA splicing through other mechanism(s).

### U2AF1-Promoter R-Loop Interaction Enhances the Occupancy of RNA Polymerase II in Gene Promoters

Previous studies showed that RNAPII complexes involved in transcription initiation and elongation can form distinct phase-separated condensates, where the transition from transcription initiation to elongation is regulated in part by RNAPII phosphorylation.^43,47^ Given the phase separation property of the U2AF1-R-loop complex, we posited that U2AF1 may assemble with RNAPII into phase-separated condensates in gene promoters, thereby augmenting the partition of RNAPII into transcription initiation complexes. To test the hypothesis, we conducted RNAPII ChIP-seq experiments to examine how genetic depletion of U2AF1 and DHX9 modulates the genome-wide distribution of RNAPII in HEK293T cells. Our results showed that U2AF1 depletion led to diminished RNAPII occupancy in promoter regions, which is accompanied by a slightly increased occupancy of RNAPII in gene bodies (Figure 6D). On the other hand, depletion of DHX9 resulted in an elevated occupancy of RNAPII in promoter regions, indicating that U2AF1 and promoter R-loops play important roles in recruiting RNAPII into the transcription initiation complex (Figure 6D). We also observed a rise in RNAPII occupancy in coding regions in DHX9-depleted cells, albeit to a lesser extent than that in promoter regions (Figure 6D), which might arise from DHX9’s activity in unwinding low levels of R-loops in the coding regions. In addition, our ChIP-seq density analysis indicated that genetic depletions of U2AF1 and DHX9 modulate the densities of RNAPII in promoter and coding sequence (CDS) regions (Figure 6E), which is consistent with the aforementioned results from the metagene profile analysis. Furthermore, we validated our ChIP-seq results for several genes, including *SRRM1*, *SETX*, *LENG8*, and *CHEK2*, by ChIP-qPCR analyses (Figure S11B). Representative Integrative Genomics Viewer (IGV) plots depicted the enrichment of RNAPII in the promoter region of the *LENG8* gene in these cell lines (Figure S11C). Together, these results suggest that U2AF1-R-loop interaction contributes to the recruitment of RNAPII to gene promoters, thereby diminishing its partition into the transcription elongation complex and attenuating cotranscriptional pre-mRNA splicing.

## Discussion

R-loops and G4s, two important types of noncanonical nucleic acid structures, are known to exist throughout the human genome and play crucial roles in many cellular processes.^1,48^ In addition, multiple studies revealed a GC skew in the displaced ssDNA in R-loops, suggesting formation of the G4 structure on the displaced ssDNA as well as the colocalization of R-loops with DNA G4s in the human genome.^3,4^ Several recent studies also documented a positive association between R-loops and G4s in gene regulation and genome stability maintenance.^13,14^ Thus, a comprehensive assessment about cellular proteins recognizing both R-loops and DNA G4s may provide insights into the functional interplay of R-loops and DNA G4s in gene regulation.

In this study, we presented a bioinformatic approach, relying on the overlapping analysis of transcription factor ChIP-seq data sets with R-ChIP-seq and BG4-ChIP-seq data sets, to identify R-loop- and DNA G4-binding proteins. Our bioinformatic analysis revealed a large number of candidate R-loop- or G4-binding proteins as well as proteins recognizing both types of nucleic acid secondary structures (Figure 1B). Interestingly, we found that the binding sites of many splicing factors (U2AF1, SRSF1, etc.) are highly colocalized with chromatin loci enriched with R-loops and G4 structures.

Our bioinformatic analysis revealed that U2AF1 ChIP-seq peaks are highly enriched at R-loop and G4 peak centers, where the co-occupancy of U2AF1, R-loop, and G4 occurs primarily in promoter regions, indicating that U2AF1’s occupancy in gene promoters arises from its interactions with these nucleic acid structures (Figure 1 and Figures S1 and S2). We also demonstrated the direct binding of purified U2AF1 toward R-loop and DNA G4 probes at low-nM binding affinities through fluorescence anisotropy and EMSA measurements (Figure 2 and Figures S3, S4, and S6). Moreover, the high binding affinity of U2AF1 for the G4 DNA probe entails its G4 folding (Figure S4). These results lent evidence to support U2AF1 as a R-loop-binding protein, and our results also suggested the enhancement of this binding by the presence of G4 in the displaced ssDNA. In this vein, Sims et al.^49^ observed that H3K9me3 enables the recruitment of U2 snRNPs to transcription start sites to facilitate cotranscriptional pre-mRNA splicing. Chen et al.^3^ later showed the enrichment of R-loop structures in promoter regions. Our revelation of U2AF1’s ability to directly and strongly bind with R-loops establishes an important biochemical origin for the occupancy of U2AF1 and by extension, the U2 snRNP complex on the promoters of actively transcribed genes. Nonetheless, we cannot formally exclude the possibility that other mechanisms may also contribute to U2AF1’s occupancy at gene promoters.

We also demonstrated that U2AF1-R-loop binding promotes LLPS of U2AF1. In particular, U2AF1 protein exhibits a weak ability to undergo LLPS on its own, and this ability can be markedly enhanced by the addition of R-loops or a crowding agent (Figure 4). Further investigations revealed that only R-loops, but no other nucleic acid structures examined, i.e., duplex DNA, RNA/DNA hybrid, or ssRNA, can stimulate the phase separation of U2AF1, and G4-R-loops exert a more pronounced effect on promoting droplet formation than M4-R-loops (Figure 4). Intermolecular G4s have been shown to facilitate the formation of multimeric complexes and trigger phase separation.^50^ It will be important to examine, in the future, the contributions of such intermolecular G4 structures to the phase separation of the U2AF1-G4-R-loop complex.

To gain additional insights into the phase separation property of the U2AF1 protein, we conducted a series of in vitro and in cellulo experiments to examine systematically the property of U2AF1 droplets. The results showed that U2AF1 undergoes phase separation in vitro and in cells, and the C-terminal RS domain of U2AF1, hydrophobic and electrostatic interactions contribute to its droplet formation (Figures 4 and Figure S9). Guo et al.^43^ showed that RNAPII with serine 2 in the C-terminal domain being phosphorylated assembles into phase-separated condensates with splicing factors. Our finding that R-loops can stimulate the LLPS of U2AF1 suggests a role of R-loops in the phase separation of the phosphorylated form of RNAPII and other splicing factors. Our RNAPII ChIP-seq results revealed that U2AF1 promotes the occupancy of RNAPII in gene promoters, suggesting that U2AF1-R-loop interactions may enhance the partition of RNAPII into transcription initiation condensates, thereby impairing its partition into transcription elongation/splicing condensates (Figure 6D,E). Hence, U2AF1-R-loop interactions may modulate mRNA splicing by influencing RNAPII’s partition into different phase-separated condensates that are involved with transcription initiation and pre-mRNA splicing.

A previous study indicated that cotranscriptional splicing may occur in subnuclear membrane-less compartments where transcription and RNA processing machineries are highly concentrated.^51^ Based on existing literature and the results from this study, we propose a model where R-loop may act as a molecular scaffold for assembling splicing factors into macromolecule complexes to enable cotranscriptional splicing, and such an assembly is guided by the LLPS principle (Figure 6F). On the other hand, excess R-loop in cells may also interfere with efficient interactions of cellular proteins with DNA and/or RNA. Indeed, our results showed that increased R-loops at promoter regions, arising from genetic depletion of DHX9, enhance the association of U2AF1 with gene promoters. Moreover, this augmented interaction with promoters impairs U2AF1’s engagement with the 3′ splicing site, thereby modulating RNA splicing.

In summary, we revealed, for the first time, U2AF1 as an R-loop- and DNA G4-binding protein, which provided a biochemical rationale for its enrichment at gene promoters. We also demonstrated that the R loop stimulates the phase separation of U2AF1, which is enhanced by the G4 structure in the displaced ssDNA in the R-loops. Additionally, the phase separation property of U2AF1 requires its C-terminal RS domain and is promoted by two zinc finger domains. Our data also suggested the functions of U2AF1-R-loop interaction in the coordination of cotranscriptional splicing through modulating the partition of RNAPII in phase-separated condensates that are involved in transcription initiation and transcription elongation/pre-mRNA splicing. It will be important to determine the high-resolution structure of the U2AF1-R-loop complex, which will offer molecular-level details about how U2AF1 recognizes this unique nucleic acid structure. In addition, it will be interesting to examine, in the future, if the findings made for U2AF1 could be extended to other proteins, especially those splicing factors, for example, SRSF1, PRPF4, and NONO, that also display high frequencies of co-occupancy with R-loops and G4 structures in chromatin.

## Materials and Methods

### Cell Culture

HEK293T cells (ATCC, Manassas, VA) were cultured in Dulbecco’s modified Eagle’s medium (DMEM, Life Technologies) containing 10% fetal bovine serum (Invitrogen) and 1% penicillin and streptomycin (Invitrogen), and the cells were maintained at 37 °C in an incubator containing 5% CO_2_.

### Bioinformatic Analysis

ChIP-seq data were retrieved from the ENCODE portal under assay title “TF ChIP-seq” and biosample classification “K562”. A total of 322 experimental results (Tables S1 and S2) were downloaded, and the IDR thresholded narrowpeak files were employed for overlapping analysis. R-ChIP-seq and G4-ChIP-seq data of K562 cells were retrieved from GEO with the accession numbers of GSE97072^3^ and GSE107690,^9^ respectively. Bedtools^52^ intersect was employed for overlapping analysis with the option “-wa -u”. The overlapping percentage was calculated as (no. of overlapped peaks)/(total no. of peaks for the target protein) × 100%. Signal enrichment was analyzed by using bwtool.^53^

### Purification of Recombinant Proteins

The plasmid for expressing recombinant His_6_-U2AF1 was constructed by first amplifying the *U2AF1* gene from a cDNA library and ligating it to the *Bam*HI and *Xho*I sites of the pET30a vector. The pET28a-EGFP-U2AF1 plasmid for expressing recombinant His_6_-EGPF-U2AF1 was constructed by replacing the CDS of the *CNA35* gene in the pET28a-EGFP-CNA35 plasmid (Addgene, #61603) with that of the *U2AF1* gene. For truncated His_6_-tagged U2AF1 proteins, the corresponding CDSs were amplified by PCR and inserted into the pET30a vector. The sequences of the plasmids were confirmed by Sanger sequencing.

The plasmids were transformed into competent Rosetta (DE3) pLysS *Escherichia coli cells*, and protein expression was induced by incubating cells with 0.5 mM isopropyl β-D-1-thio-galactopyranoside (IPTG, Sigma) at 16 °C for 20 h. The cells were subsequently harvested by centrifugation and lysed by sonication in a lysis buffer (20 mM Tris, pH 8.0, 1 M NaCl, 0.5 M urea, 25 mM imidazole, 10 mM β-mercaptoethanol, 10% glycerol, and 1 mM phenylmethylsulfonyl fluoride). After centrifugation at 10,000*g* for 15 min, the supernatant was filtered by using a 4.5 μm syringe filter and subsequently subjected to protein purification with a HisTrap HP column (1 mL, Cytiva) following the manufacturer’s recommended procedures. Protein purity was verified by SDS-PAGE analysis, quantified by Quick Start Bradford Protein Assay kit (Bio-Rad), and used immediately or stored at −80 °C until use.

### In Vitro Binding Assays

Fluorescently labeled DNA or RNA probes (500 nM, Integrated DNA Technologies, Table S3) were dissolved in an RNase-free buffer, which contained 10 mM Tris-HCl (pH 7.5), 100 mM KCl, and 0.1 mM EDTA. The probes were annealed by heating the solution to 95 °C for 5 min, followed by cooling slowly to room temperature over 3 h.

Fluorescence anisotropy-based binding assays were performed with 10 nM probes and the indicated concentrations of recombinant U2AF1 protein in a 60-μL binding buffer containing 10 mM Tris-HCl (pH 7.5), 1 mM EDTA, 100 mM KCl, 0.1 mM DTT, and 10 μg/mL BSA. After a 30 min incubation on ice, fluorescence anisotropy was recorded on a Horiba QuantaMaster-400 spectrofluorometer (Photon Technology International), with the excitation and emission wavelengths being set at 550 and 580 nm, respectively. The instrument G factor was determined prior to anisotropy measurements, and the *K*_d_ values were calculated with GraphPad Prism 8 software using nonlinear regression for curve fitting with the one-binding-site model.

EMSA was performed with 10 nM probes and various concentrations of recombinant U2AF1 protein in a 10-μL binding buffer containing 10 mM Tris-HCl (pH 8.0), 1 mM EDTA, 100 mM KCl, 0.1 mM DTT, and 10 μg/mL BSA. After a 30 min incubation on ice, the protein–nucleic acid complexes were separated from free probes on a 6% native polyacrylamide gel using 1× TAE (40 mM Tris-acetate, pH 8.0, 2 mM EDTA) by electrophoresis at 120 V for 15 min, and the gel was imaged using a Typhoon PhosphorImager (GE).

### CD Spectroscopy

The CD spectra for annealed c*MYC* G4, U2AF1 protein, and a mixture of *cMYC* G4 and U2AF1 protein (at 5 μM each) in a buffer (10 mM Tris-HCl, pH 8.0, 100 mM KCl, and 1 mM EDTA) were acquired in the wavelength range of 200–320 nm on a Jasco-815 spectropolarimeter. The CD spectra for G4-R-loop and M4-R-loop probes were recorded in the wavelength range 170–320 nm.

### In Vitro Phase Separation Assay

For in vitro phase separation assay of U2AF1 protein, equal amounts of recombinant EGFP-U2AF1 (0.5 μM) and unlabeled U2AF1 (0.5 μM) were mixed in a binding buffer (10 mM Tris-HCl, pH 7.5, 1 mM EDTA, 100 mM KCl, 0.1 mM DTT, and 10 μg/mL BSA) containing 10% dextran. For nucleic-acid-induced phase separation of U2AF1 in vitro, a 100 nM fluorescently labeled nucleic acid probe was incubated with 0.5 μM unlabeled U2AF1 in a binding buffer with or without 10% dextran. After incubation on ice for 30 min, the mixture was immediately loaded onto a glass slide and covered with a cover glass (Thorlabs). Fluorescence and DIC microscopy images were recorded on a Zeiss 880 Upright Confocal microscope with a 40× oil lens.

### FRAP

FRAP assay was performed on a Zeiss 880 Upright Confocal microscope with a 40× oil lens. Droplets with sizes of ∼2–3 μm were chosen for photobleaching, where the droplets were initiated with a 488 nm laser at maximum intensity after 3 scans and it was stopped when the intensity drops to 50%. Fluorescence recovery was recorded every 1 s for 135 s after photobleaching.

### Live-Cell Imaging and Fixed-Cell Imaging

For live-cell imaging, HEK293T cells were plated in 35 mm glass-bottom dishes (MatTek) in phenol-free complete DMEM medium and transfected with mCherry-U2AF1 plasmid (Addgene, #84017). After 24 h, the cells were treated with 10% 1,6-hexanediol and immediately imaged on a Zeiss 880 Inverted confocal microscope in a 37 °C humidified chamber with 5% CO_2_ using a 40× oil lens.

For fixed-cell imaging, cells were plated on cover glasses in a 12-well plate and transfected with the indicated plasmids. After 24 h, the cells were washed once with PBS-TX (PBS containing 0.1% Triton X-100), followed by fixing in ice-cold methanol at rt for 15 min. After washing twice with PBS-TX, the nuclei were stained with 1 μg/mL DAPI (Sigma) in PBS-TX at rt in the dark for 5 min. After washing twice with PBS-TX, cover glasses were mounted onto microscope slides with the cell side down. The images were acquired on a Zeiss 880 inverted confocal microscope using a 40× oil lens.

### Lentivirus Production and Transduction

HEK293T cells were seeded in a 10 cm dish at 30% confluence 1 day prior to transfection with 4 μg of pLKO.1 puro plasmid (Addgene no. 8453) for shRNA expression, 1 μg of pLTR-G (Addgene no. 17532) envelope plasmid, and 3 μg of pCMV-dR8.2 dvpr (Addgene no. 8455) package plasmid together with 40 μL of PolyFect transfection reagent (Qiagen). The medium was replaced 12 h after transfection. After 48 h, viral particles were harvested from the culture medium and filtered with a 0.45-μm sterile filter (Millipore). Cells were transduced with a lentivirus for 48 h and subsequently screened with 1.0 μg/mL puromycin. The shRNA sequences are listed in Table S4.

### RT-PCR

Total RNA was extracted using an Omega Total RNA Kit I (Omega) and quantified. Reverse transcription was conducted by using M-MLV Reverse Transcriptase (Promega) to obtain the cDNA library. PCR was performed using DreamTaq Green PCR Master Mix (Thermo Fisher Scientific), and the resulting PCR products were separated on a 3% agarose gel using 1× TAE buffer (40 mM Tris-acetate, pH 8.0, and 2 mM EDTA). Electrophoresis was performed at 130 V for 30 min, and the gel was imaged with an Odyssey Imaging System (LI-COR Biosciences). The primers for RT-PCR are shown in Table S5.

### ChIP-qPCR and ChIP-seq

R-ChIP experiments and U2AF1-ChIP were performed with HEK293T cells stably expressing V5-tagged catalytically inactive RNase H1 mutants (i.e., RH1^D210N^ or RH1^WKKD^ protein)^3^ and Flag-tagged U2AF1 protein, respectively. RNAPII-ChIP was conducted with control HEK293T cells and the same cells with U2AF1 or DHX9 being knocked down. Briefly, ∼1 × 10^7^ cells were cross-linked with 1% formaldehyde at room temperature with gentle shaking for 12 min and quenched with 125 mM glycine for 10 min. After washing with ice-cold PBS buffer for three times, the cells were resuspended in a lysis buffer (10 mM Tris-HCl, pH 8.0, 10 mM NaCl, 0.5% NP-40, and protease inhibitor cocktail) at 4 °C on a rotator for 20 min. After centrifugation at 3000*g* for 3 min, the pellets were resuspended in RIPA buffer (50 mM Tris-HCl, pH 8.0, 150 mM NaCl, 2 mM EDTA, 1% NP-40, 0.5% sodium deoxycholate, 0.1% SDS, and protease inhibitor cocktail) and rotated at 4 °C for 20 min, followed by a brief sonication using Qsonica Sonicator q125 (42% amplitude, 10s on/10s off, 80 s). After incubation for another 20 min, the cell lysate was sonicated with a Covaris S220 Sonicator for 6 min with a peak incident power of 140 W, a duty cycle of 10.1%, and 200 cycles per burst at 4 °C. After centrifugation at 16,000*g* for 10 min, 50 μL of supernatant was taken out and used as the “Input” sample, and the rest was subjected to IP experiment. Protein A/G beads (Santa Cruz, sc-2003) conjugated with anti-V5 antibody (ProteinTech, 14440–1-AP) and POLR2A antibody (Thermo Fisher Scientific, MA1–26249) were used for the R-ChIP and RNAPII-ChIP experiments, respectively. Anti-Flag M2 Affinity Gel (Sigma, A2220) was employed for the U2AF1-ChIP experiments, where the cells transfected with empty vector were used as a negative control.

The IP experiment was conducted at 4 °C overnight on a rotator. On the next day, beads were washed twice with low-salt washing buffer (20 mM Tris-HCl, pH 8.0, 150 mM NaCl, 2 mM EDTA, 1% Triton X-100, and 0.1% SDS), twice with high-salt washing buffer (20 mM Tris-HCl, pH 8.0, 500 mM NaCl, 2 mM EDTA, 1% Triton X-100, and 0.1% SDS), twice with LiCl buffer (10 mM Tris-HCl, pH 8.0, 0.25 M LiCl, 1 mM EDTA, 1% NP-40, and 1% sodium deoxycholate), and once with TE buffer. The protein–chromatin complex was eluted with 120 μL of elution buffer (1% SDS and 100 mM NaHCO_3_) at 65 °C for 30 min on a thermomixer (1000 rpm). After centrifugation, the supernatant was transferred to a new tube, followed by adding 4.8 μL of 5 M NaCl and 2 μL of RNase A. After reverse cross-linking and RNA removal at 65 °C for over 8 h, proteins were digested with 2 μL of proteinase K at 60 °C for 1 h. Subsequently, DNA was purified using ChIP DNA Clean & Concentrator (Zymo Research, D5205). For “Input” samples, reverse cross-linking, RNA digestion, protein digestion, and DNA purification were conducted in the same way as the “IP” samples.

The recovered DNA fragments were subjected to qPCR analysis and DNA library preparation. The qPCR was performed using a Luna Universal qPCR Master Mix (NEB) on the CFX96 RT-qPCR detection system (Biorad). Primers used for ChIP-qPCR are listed in Table S6. The DNA-sequencing library was prepared using the NEBNext Ultra DNA Library Prep Kit for Illumina (NEB) following the manufacturer’s instructions. The purified DNA libraries were subsequently quantified using an Agilent 2100 Bioanalyzer and multiplexed for sequencing on an MGI-seq 2000 instrument (BGI, China) with 100-bp paired-end sequencing.

Raw reads were aligned to human hg38 reference genome using Bowtie2 (v2.4.5)^54^ in default setting. Genome coverage bigwig files for IGV visualization were generated by deeptools (v3.5.1)^55^ bamCoverage using “RPKM” for the normalization. Peaks were generated by macs2 (v2.2.7.1)^56^ callpeak. Metagene profile was generated by deeptools (v3.5.1) computeMatrix and plotProfile. ChIP-seq density was calculated by Homer (v4.8.2)^57^ analyzeRepeats. Boxplots were generated with GraphPad Prism 9.4.

### CLIP-qPCR

CLIP experiment was performed with cells expressing either empty vector or Flag-tagged U2AF1 protein. Briefly, cells in 15 cm dishes were cultured to 50% confluence at the time of transfection. At 24 h following transfection, the medium was replaced with PBS, and the sample was irradiated with 400 mJ/cm^2^ at 254 nm on ice. After irradiation, the cells were collected and resuspended in nuclear lysis buffer containing 2 mL of PBS, 2 mL of nuclear isolation buffer (1.28 M sucrose, 40 mM Tris-HCl, pH 7.5, 20 mM MgCl_2_, and 4% Triton X-100), and 6 mL of diethyl pyrocarbonate (DEPC) water and incubated at 4 °C on a rotator for 20 min. After centrifugation at 2500*g* for 5 min, the pellets were resuspended in 0.8 mL RIP buffer (150 mM KCl, 50 mM Tris-HCl, pH 7.5, 5 mM EDTA, 0.5 mM DTT, 0.5% NP-40, protease inhibitor cocktail, and RNase inhibitor) and rotated at 4 °C for 15 min, followed by a brief sonication using Qsonica Sonicator q125 (42% amplitude, 10 s on/10 s off, 60 s) and incubating for another 15 min. After centrifugation at 16,000*g* for 10 min, 20 μL of supernatant was taken out and used as the “Input” sample, and the rest was subjected to the IP experiment with anti-Flag beads.

The IP experiment was conducted at 4 °C for 3 h on a rotator. After washing three times with RIP buffer and once with PBS, the beads were incubated with 10 units of DNase I in 100 μL of PBS for 15 min at 37 °C on a thermomixer. After adding 700 μL of PBS, the beads were centrifuged to remove the buffer, followed by incubating in 100 μL of PBS supplemented with 0.1% SDS and 0.5 mg/mL proteinase K for 15 min at 37 °C on a thermomixer. After centrifugation at 10000*g* for 5 min, the supernatant was collected into a new tube as “IP” samples. RNAs in both “Input” and “IP” samples were extracted with TRIzol reagent. Reverse transcription was conducted using M-MLV Reverse Transcriptase (Promega) with gene-specific primers as well as dT_18_ oligo, and the resulting cDNA libraries were subjected to qPCR analysis. Primers used for CLIP-qPCR are listed in Table S7.

## Data Availability

The RNAPII-ChIP-seq data has been deposited into the NCBI GEO database with accession number GSE230254.

## Supporting Information Available

Results obtained from overlapping analysis, lists of primer and shRNA sequences, EMSA and fluorescence anisotropy for monitoring the binding of U2AF1 with different nucleic acid substrates, and phase separation results (PDF)
