# Supplementary material for: Promoter R-Loops Recruit U2AF1 to Modulate Its Phase Separation and RNA Splicing
Source: J Am Chem Soc. 2023 Sep 21;145(39):21646–60. doi: 10.1021/jacs.3c08204 (PMC10557143; doi:10.1021/jacs.3c08204)

Supplementary Materials for

"Promoter R-loops Recruit U2AF1 to Modulate Its Phase Separation and RNA Splicing"

Xiaomei He[1], Jun Yuan[2], Zi Gao[1], and Yinsheng Wang[1,2,*]

[1]Department of Chemistry and [2]Environmental Toxicology Graduate Program, University of California Riverside, Riverside, CA 92521-0403

*To whom correspondence should be addressed: yinsheng@ucr.edu

Table of Contents

Table S1. Overlapping percentages of BG4-ChIP-seq and ChIP-seq peaks.

Table S2. Overlapped percentages of R-ChIP-seq and ChIP-seq peaks.

Table S3. A list of DNA and RNA probes used for *in vitro* assays.

Table S4. A list of shRNA sequences for the stable knockdown experiments.

Table S5. A list of primers for RT-PCR experiments.

Table S6. A list of primers for ChIP-qPCR experiments.

Table S7. A list of primers for CLIP-qPCR experiments.

Figure S1. U2AF1 is predominantly enriched in promoter regions in human cells.

Figure S2. The binding preferences of U2AF1 protein are recapitulated in HepG2 cells.

Figure S3. Fluorescence anisotropy for assessing the binding of recombinant U2AF1 protein toward G4, M4, and dsDNA probes.

Figure S4. EMSA for examining the binding of recombinant U2AF1 protein toward G4, and M4 DNA probes.

Figure S5. Circular dichroism (CD) spectroscopy for monitoring G4 and R-loop structures.

Figure S6. EMSA for assessing the interaction between full-length U2AF1 protein and R-loop probes.

Figure S7. U2AF1-Rloop interaction elicits phase separation of U2AF1 protein *in vitro*.

Figure S8. U2AF1 phase separation could be enhanced by dextran and R-loop.

Figure S9. The material properties of U2AF1 droplets.

Figure S10. The ZF domains contribute to the phase separation of U2AF1.

Figure S11. R-ChIP-qPCR and RNAPII-ChIP-seq results.

Table S1. Overlapping percentages between BG4-ChIP-seq peaks and ChIP-seq peaks for 322 proteins.

Datasets	Genes	Total Peaks	Overlap Peaks	Overlap Percentages (%)
ENCFF553GPK	SP1	12092	6725	55.62
ENCFF071GJH	RBM34	1084	595	54.89
ENCFF410RJD	NRF1	4436	2306	51.98
ENCFF552PTK	FUS	2876	1469	51.08
ENCFF724DNU	POU5F1	2091	1033	49.40
ENCFF075BRS	GTF2F1	4867	2327	47.81
ENCFF709DMO	PRPF4	6792	3217	47.36
ENCFF211TTD	NONO	5906	2701	45.73
ENCFF895RNS	ATF3	1204	545	45.27
ENCFF823RYG	THAP1	4740	2144	45.23
ENCFF029RBI	SREBF1	2848	1283	45.05
ENCFF503XOK	U2AF1	4430	1973	44.54
ENCFF285TMA	FIP1L1	10437	4633	44.39
ENCFF924GPK	ATF1	3550	1575	44.37
ENCFF792SXS	SRSF1	1276	556	43.57
ENCFF715WGN	ELK1	3793	1630	42.97
ENCFF730QQD	SMAD2	133	57	42.86
ENCFF257NOX	SIN3B	8979	3806	42.39
ENCFF304PGC	KDM4B	7999	3390	42.38
ENCFF588GNU	CEBPZ	1497	634	42.35
ENCFF813TRY	DEAF1	3610	1528	42.33
ENCFF981ISM	PHF8	29363	12389	42.19
ENCFF863WFD	GMEB1	10003	4195	41.94
ENCFF091TCH	RBM15	284	119	41.90
ENCFF675PHY	MYC	5139	2085	40.57
ENCFF469KAH	SIN3A	13197	5240	39.71
ENCFF833ZNA	HNRNPLL	15057	5950	39.52
ENCFF230UNT	NR2C2	571	224	39.23
ENCFF139FMU	HCFC1	13052	4940	37.85
ENCFF159OUK	ETS1	13775	5203	37.77
ENCFF788YHU	MBD2	15309	5672	37.05
ENCFF156DSB	TARDBP	8496	3147	37.04
ENCFF674KVR	KLF1	9083	3363	37.03
ENCFF956MGE	PCBP1	10978	4039	36.79
ENCFF493DCP	MAX	4112	1511	36.75
ENCFF118GMS	CREB3L1	17197	6289	36.57
ENCFF807FNB	KDM5B	22368	8141	36.40

Datasets	Genes	Total Peaks	Overlap Peaks	Overlap Percentages (%)
ENCFF453MMH	KLF13	9096	3259	35.83
ENCFF107SJD	POLR2A	23711	8490	35.81
ENCFF068IGH	MXI1	9081	3249	35.78
ENCFF111ICL	ZHX1	3294	1156	35.09
ENCFF854WAP	HNRNPL	8047	2820	35.04
ENCFF942VGF	RBBP5	24754	8670	35.02
ENCFF053HPQ	E2F1	7957	2781	34.95
ENCFF971YTJ	SMAD5	21973	7661	34.87
ENCFF074OAM	YY1	16564	5720	34.53
ENCFF120PGJ	SMAD1	8465	2915	34.44
ENCFF537RNU	SAFB	6171	2096	33.97
ENCFF938ZPZ	E2F7	2624	887	33.80
ENCFF742ZHT	SIX5	3458	1162	33.60
ENCFF085GHX	E2F6	16349	5432	33.23
ENCFF625QHR	NEUROD1	14155	4652	32.86
ENCFF370YGS	TBP	22063	7239	32.81
ENCFF362JHQ	TFDP1	22966	7420	32.31
ENCFF564KIU	GTF2E2	16064	5136	31.97
ENCFF227HVU	ZBTB11	4058	1287	31.72
ENCFF383IEP	SAP30	14282	4529	31.71
ENCFF067KCK	SKIL	18817	5965	31.70
ENCFF392INU	ZBED1	6053	1906	31.49
ENCFF092DLN	RBM14	2989	938	31.38
ENCFF118ECK	REST	10419	3267	31.36
ENCFF110GDP	ZNF282	9595	2984	31.10
ENCFF889QYR	SUZ12	2737	849	31.02
ENCFF321LMQ	NFRKB	6938	2151	31.00
ENCFF925GLW	E2F8	11411	3535	30.98
ENCFF053PDX	PHF20	14101	4348	30.83
ENCFF732ZNB	TCF7L2	2094	645	30.80
ENCFF568ZPW	UBTF	9069	2782	30.68
ENCFF070ZTX	BCLAF1	5586	1711	30.63
ENCFF910JTR	ZFX	27194	8310	30.56
ENCFF409SDU	RNF2	4717	1434	30.40
ENCFF669VJB	ZNF639	15628	4727	30.25
ENCFF736XUU	TCF7	5501	1659	30.16
ENCFF627UZT	MNT	22216	6668	30.01
ENCFF034SWM	TBL1XR1	5774	1725	29.88
ENCFF470CSE	CBX1	14593	4350	29.81
ENCFF479SOJ	RB1	24328	7143	29.36

Datasets	Genes	Total Peaks	Overlap Peaks	Overlap Percentages (%)
ENCFF374EFU	RUNX1	3797	1113	29.31
ENCFF528SIV	ZC3H11A	2489	725	29.13
ENCFF489EME	GABPA	16493	4791	29.05
ENCFF792CKI	TAF7	4634	1342	28.96
ENCFF821QOS	POLR2B	21193	6087	28.72
ENCFF661EUX	RFX5	2631	754	28.66
ENCFF538ACI	RBFOX2	57071	16195	28.38
ENCFF708IYN	XRCC5	12294	3450	28.06
ENCFF669MJX	HDAC1	16391	4547	27.74
ENCFF738XMN	SUPT5H	31169	8638	27.71
ENCFF553IUR	ASH1L	6135	1691	27.56
ENCFF280AIK	MTA3	26199	7210	27.52
ENCFF130JVF	BRD4	9337	2552	27.33
ENCFF316IYH	ZMIZ1	1078	293	27.18
ENCFF746TUQ	DDX20	9274	2507	27.03
ENCFF605XYC	MITF	7517	1995	26.54
ENCFF137TCL	NR4A1	4795	1272	26.53
ENCFF133TSU	ELF1	20718	5458	26.34
ENCFF432HRL	TAF9B	15025	3942	26.24
ENCFF051FNO	PML	20683	5356	25.90
ENCFF752VQB	RLF	8951	2310	25.81
ENCFF794IRP	AGO1	11400	2937	25.76
ENCFF167CQF	EP400	34365	8813	25.65
ENCFF084KHS	POLR2H	24342	6151	25.27
ENCFF559ODJ	IRF2	8579	2157	25.14
ENCFF382CUT	ILF3	3450	857	24.84
ENCFF797VEK	ZZZ3	2532	623	24.61
ENCFF928BHE	DIDO1	6652	1635	24.58
ENCFF746OAD	CSDE1	5053	1234	24.42
ENCFF215JWS	PHF21A	7062	1696	24.02
ENCFF068MGV	NKRF	17206	4130	24.00
ENCFF209DVO	ZBTB2	16460	3916	23.79
ENCFF150ZBY	ZNF830	4097	967	23.60
ENCFF841RMH	PBX2	3908	920	23.54
ENCFF996OEL	NCOA6	4096	963	23.51
ENCFF433ERT	VEZF1	46848	10973	23.42
ENCFF455OST	HNRNPK	11085	2589	23.36
ENCFF535YSH	HNRNPH1	287	67	23.34
ENCFF599CIT	GABPB1	30600	7132	23.31
ENCFF388KGW	GATAD2B	13588	3144	23.14

Datasets	Genes	Total Peaks	Overlap Peaks	Overlap Percentages (%)
ENCFF413LLO	POLR2G	58640	13565	23.13
ENCFF657LFS	CCAR2	511	118	23.09
ENCFF219NIA	ZNF740	8088	1865	23.06
ENCFF752EMN	SETDB1	4667	1056	22.63
ENCFF060MMW	POLR2AphosphoS5	30051	6790	22.59
ENCFF346AOR	ZBTB7A	28731	6491	22.59
ENCFF871CWZ	E2F5	19099	4310	22.57
ENCFF736FGN	HDAC6	1490	336	22.55
ENCFF563WUP	TOE1	21449	4817	22.46
ENCFF616FCV	ELF4	18273	4102	22.45
ENCFF015FXW	ZNF407	3359	747	22.24
ENCFF566PEY	HDAC8	6892	1529	22.19
ENCFF030QYO	SRSF7	254	56	22.05
ENCFF584QFY	ETV6	2509	545	21.72
ENCFF744HVD	USF2	3621	786	21.71
ENCFF937QYU	RBM17	51	11	21.57
ENCFF951KHS	POLR2AphosphoS2	9579	2063	21.54
ENCFF146GZZ	ZBTB33	3360	722	21.49
ENCFF430IPX	ZNF395	25070	5379	21.46
ENCFF772QLT	ETS2	905	193	21.33
ENCFF600KEF	BMI1	2850	601	21.09
ENCFF987UBO	ZNF197	10709	2256	21.07
ENCFF108MQY	EGR1	27406	5714	20.85
ENCFF683WRK	E4F1	35526	7400	20.83
ENCFF382IYX	ZBTB5	2928	609	20.80
ENCFF630HGY	HDAC2	9026	1875	20.77
ENCFF951BFN	ATF7	42855	8864	20.68
ENCFF560UGR	ZKSCAN1	13641	2803	20.55
ENCFF987DPX	RBM22	12852	2608	20.29
ENCFF048BKZ	SRSF3	1805	365	20.22
ENCFF669RLC	NUFIP1	2413	484	20.06
ENCFF670POK	NBN	19293	3835	19.88
ENCFF689HWD	LEF1	4000	786	19.65
ENCFF647RGI	NR2F6	2399	470	19.59
ENCFF621FPC	HDGF	10280	2013	19.58
ENCFF295YRT	GATAD2A	11781	2300	19.52
ENCFF283UWH	ZNF148	23554	4575	19.42
ENCFF331PZL	COPS2	31	6	19.35
ENCFF020XNM	SNIP1	2166	418	19.30
ENCFF756TIY	RBM39	2856	551	19.29

Datasets	Genes	Total Peaks	Overlap Peaks	Overlap Percentages (%)
ENCFF588QRH	ESRRA	29519	5695	19.29
ENCFF375SIS	ID3	18808	3617	19.23
ENCFF176MDV	SAFB2	63	12	19.05
ENCFF547PES	TAF15	798	152	19.05
ENCFF451LLC	ZNF83	11150	2119	19.00
ENCFF782PIZ	CTBP1	46794	8811	18.83
ENCFF058KJG	FOXK2	21477	4035	18.79
ENCFF063NIH	L3MBTL2	50803	9508	18.72
ENCFF324ELP	CREM	38537	7156	18.57
ENCFF835NOD	PTBP1	4896	899	18.36
ENCFF407STM	ZSCAN29	4540	807	17.78
ENCFF154IVU	BHLHE40	27808	4891	17.59
ENCFF147IAH	MCM2	222	39	17.57
ENCFF516ZEQ	MYNN	15432	2707	17.54
ENCFF898CGY	NCOR1	7181	1258	17.52
ENCFF525XXS	KHSRP	4094	713	17.42
ENCFF773XPT	ZNF644	12950	2254	17.41
ENCFF190CGV	JUN	21522	3728	17.32
ENCFF045AOZ	ZC3H8	11058	1910	17.27
ENCFF058ZHN	MTA1	16037	2768	17.26
ENCFF961ADR	NR3C1	1509	259	17.16
ENCFF920CRL	GTF2A2	5617	954	16.98
ENCFF674XTY	AFF1	9039	1517	16.78
ENCFF224YBQ	RCOR1	6705	1121	16.72
ENCFF957CVJ	FOXJ2	15359	2566	16.71
ENCFF080IBW	MTA2	17005	2811	16.53
ENCFF486QCV	SMARCA4	3351	552	16.47
ENCFF328OCA	JUNB	5438	888	16.33
ENCFF469ZBB	NR2C1	15072	2449	16.25
ENCFF313PVP	ETV1	22167	3560	16.06
ENCFF113LBV	KAT8	1309	210	16.04
ENCFF345IHK	ZNF589	20982	3362	16.02
ENCFF649OPX	ZNF24	13998	2216	15.83
ENCFF972OBR	NFATC3	11347	1762	15.53
ENCFF614IBI	HLTF	10568	1616	15.29
ENCFF652ZEN	SFPQ	237	36	15.19
ENCFF066OJB	CBX5	6985	1060	15.18
ENCFF821MKR	EP300	3056	455	14.89
ENCFF217LXF	SMARCB1	2393	356	14.88
ENCFF355OYM	MLLT1	10657	1578	14.81

Datasets	Genes	Total Peaks	Overlap Peaks	Overlap Percentages (%)
ENCFF509ZLE	GATA1	4603	678	14.73
ENCFF671ZGP	HES1	9121	1340	14.69
ENCFF524ZER	MGA	33583	4898	14.58
ENCFF624PTB	ZBTB40	31742	4588	14.45
ENCFF835PDO	ZFP91	13459	1944	14.44
ENCFF878SVX	ZNF507	5466	785	14.36
ENCFF528OYW	ZNF766	26392	3778	14.31
ENCFF005MBI	ZNF584	14945	2126	14.23
ENCFF386ZWO	CBX3	24848	3520	14.17
ENCFF192MEM	ZNF175	18987	2688	14.16
ENCFF405BKB	PRDM10	28414	3991	14.05
ENCFF877ZKU	YBX3	1793	247	13.78
ENCFF125GZU	SOX6	32977	4487	13.61
ENCFF637PFI	MYBL2	4912	660	13.44
ENCFF075OIT	ARNT	4251	570	13.41
ENCFF412WOK	DNMT1	2339	308	13.17
ENCFF711ZED	FOXM1	15464	1993	12.89
ENCFF775WSL	BRD9	15213	1959	12.88
ENCFF536WBT	RAD51	16146	1963	12.16
ENCFF829IEE	CREB3	10580	1253	11.84
ENCFF936WES	RELA	6523	772	11.84
ENCFF847QJI	ZNF592	38779	4567	11.78
ENCFF045CFL	ZNF274	3228	378	11.71
ENCFF110HVI	TRIM25	4898	573	11.70
ENCFF306SZL	JUND	47180	5519	11.70
ENCFF136KLM	CUX1	7061	802	11.36
ENCFF540TWL	ZNF143	29676	3365	11.34
ENCFF475CIS	ZEB2	21094	2387	11.32
ENCFF634OOU	TRIM28	16589	1863	11.23
ENCFF408RSJ	CDC5L	4916	548	11.15
ENCFF031NTF	MEF2A	6407	709	11.07
ENCFF453GKK	NFE2	2775	307	11.06
ENCFF990SFN	ZNF318	8956	989	11.04
ENCFF847ZHF	NR2F2	20217	2224	11.00
ENCFF304PQQ	LARP7	7437	815	10.96
ENCFF058VGZ	ZNF184	12442	1353	10.87
ENCFF156GHD	CC2D1A	22663	2420	10.68
ENCFF166AFQ	HINFP	6322	675	10.68
ENCFF488OTN	KLF16	17850	1884	10.55
ENCFF026BDA	BACH1	4740	500	10.55

Datasets	Genes	Total Peaks	Overlap Peaks	Overlap Percentages (%)
ENCFF729DNM	DACH1	16906	1694	10.02
ENCFF562XKK	MIER1	8019	801	9.99
ENCFF527MLG	TRIM24	31849	3161	9.92
ENCFF620FIH	BRCA1	2480	241	9.72
ENCFF775PIY	HDAC3	9985	959	9.60
ENCFF952WKN	ARHGAP35	3291	314	9.54
ENCFF335VOJ	PYGO2	2836	270	9.52
ENCFF716PXH	DPF2	23790	2263	9.51
ENCFF732IRT	PCBP2	2583	243	9.41
ENCFF169MAE	NFIC	51804	4818	9.30
ENCFF099RDJ	PKNOX1	48694	4508	9.26
ENCFF004HXL	FOSL1	12760	1178	9.23
ENCFF746GDG	NR2F1	36225	3333	9.20
ENCFF891OQP	ARID3A	11378	1030	9.05
ENCFF542DHM	MEF2D	12632	1123	8.89
ENCFF908MWB	SMARCC2	14828	1318	8.89
ENCFF187BVL	STAT5A	13167	1164	8.84
ENCFF908SNB	ZKSCAN8	11411	1001	8.77
ENCFF861ZJL	MEIS2	47442	4157	8.76
ENCFF067BCD	NCOA4	1910	161	8.43
ENCFF515GUE	CHAMP1	12909	1079	8.36
ENCFF148YMC	SMARCE1	33953	2814	8.29
ENCFF501XJP	TEAD4	35971	2968	8.25
ENCFF785QJM	PHB2	5751	469	8.16
ENCFF820GPR	RFX1	17869	1456	8.15
ENCFF872RQG	NCOA2	2393	194	8.11
ENCFF637SIR	IKZF1	47712	3790	7.94
ENCFF504PFQ	TCF12	13907	1097	7.89
ENCFF123LZK	SMC3	26590	2006	7.54
ENCFF864XZP	ZNF384	21354	1605	7.52
ENCFF497ISV	GATA2	11447	826	7.22
ENCFF649UCX	ZNF354B	4177	296	7.09
ENCFF879NTL	ARID1B	53250	3720	6.99
ENCFF700VSW	HNRNPUL1	1033	72	6.97
ENCFF839GAS	ZFP36	24500	1668	6.81
ENCFF124FTW	BCOR	41088	2771	6.74
ENCFF888CKG	SPI1	32606	2155	6.61
ENCFF852ZRK	TAL1	29476	1948	6.61
ENCFF423CWQ	ZBTB8A	610	40	6.56
ENCFF209WPT	TEAD2	4588	291	6.34

Datasets	Genes	Total Peaks	Overlap Peaks	Overlap Percentages (%)
ENCFF054XCG	KDM1A	30522	1881	6.16
ENCFF913WRW	ARID2	9561	589	6.16
ENCFF674ZQI	MCM7	1925	118	6.13
ENCFF038WQY	C11orf30	56447	3333	5.90
ENCFF992HUS	THRA	1419	82	5.78
ENCFF955QCD	TRIP13	1554	88	5.66
ENCFF582SNT	CTCF	49426	2783	5.63
ENCFF724CHN	IRF9	7452	419	5.62
ENCFF849VEO	RBM25	50554	2812	5.56
ENCFF022KBK	CEBPB	27107	1502	5.54
ENCFF859PDK	CBFA2T2	29691	1572	5.29
ENCFF341CNM	NFXL1	4624	243	5.26
ENCFF557FUM	IRF1	13777	718	5.21
ENCFF189OHQ	EHMT2	17334	901	5.20
ENCFF451AEQ	ZNF316	20293	1046	5.15
ENCFF739AJO	ADNP	21853	1040	4.76
ENCFF722RWS	SMARCA5	17172	814	4.74
ENCFF067FJF	THRAP3	4957	230	4.64
ENCFF439TJM	MAFK	27213	1249	4.59
ENCFF672ZQW	HMBOX1	29352	1334	4.54
ENCFF082DOH	CBFA2T3	49658	2193	4.42
ENCFF497OQD	FOXA1	1013	36	3.55
ENCFF192ASP	ATF2	41768	1443	3.45
ENCFF086TAD	ATF4	40114	1254	3.13
ENCFF744FLC	SNRNP70	810	24	2.96
ENCFF456USL	CEBPG	40346	1061	2.63
ENCFF119AHD	MAFF	27876	659	2.36
ENCFF491UBF	PTRF	3918	85	2.17
ENCFF143MEF	ILK	1463	28	1.91
ENCFF918BZG	PTTG1	4324	79	1.83
ENCFF366XDW	NFE2L1	6370	97	1.52
ENCFF571LUR	MAFG	41886	618	1.48
ENCFF184IOY	ZNF512	22842	296	1.30
ENCFF117XRE	ZMYM3	40933	424	1.04
ENCFF223FOF	NR0B1	110	1	0.91
ENCFF282KHW	MCM5	744	5	0.67
ENCFF583XAW	MCM3	1790	12	0.67
ENCFF932ZCO	ZNF280A	4927	33	0.67
ENCFF705SFR	TSC22D4	1556	9	0.58
ENCFF286IPW	YBX1	533	3	0.56

Datasets	Genes	Total Peaks	Overlap Peaks	Overlap Percentages (%)
ENCFF137OGC	XRCC3	1138	6	0.53
ENCFF924FYI	EWSR1	1264	3	0.24
ENCFF702DWT	U2AF2	131	0	0.00
ENCFF746IEZ	SRSF9	21	0	0.00

Table S2. Overlapping percentages between R-ChIP-seq peaks and ChIP-seq peaks for 322 proteins.

Datasets	Genes	Total Peaks	Overlap Peaks	Overlap Percentages (%)
ENCFF034CQR	TAF7	648	180	27.78
ENCFF971VJZ	RBM15	400	98	24.50
ENCFF798URE	SRSF1	1170	278	23.76
ENCFF861YKK	RBM17	47	11	23.40
ENCFF589EVD	ZBTB8A	955	223	23.35
ENCFF786WZD	DEAF1	3565	804	22.55
ENCFF785ACI	PRPF4	6788	1444	21.27
ENCFF134NMW	NONO	5818	1224	21.04
ENCFF142CPK	FUS	2947	592	20.09
ENCFF156WRH	GTF2F1	4919	982	19.96
ENCFF782GWS	RBM34	1085	213	19.63
ENCFF730URE	U2AF1	4352	814	18.70
ENCFF292JRY	HNRNPH1	315	57	18.10
ENCFF203EDM	BCLAF1	2159	388	17.97
ENCFF905VXX	TARDBP	8596	1488	17.31
ENCFF716LRI	RFX5	2725	458	16.81
ENCFF925MHB	CEBPZ	1303	218	16.73
ENCFF195ODE	ZHX1	3453	575	16.65
ENCFF883WYX	FIP1L1	10793	1784	16.53
ENCFF019PEL	ELK1	3967	653	16.46
ENCFF784FIE	NR3C1	2402	394	16.40
ENCFF985TSH	SREBF1	2865	463	16.16
ENCFF757ODD	SFPQ	228	36	15.79
ENCFF076YZO	ETS1	11558	1822	15.76
ENCFF677OWM	NFRKB	7903	1229	15.55
ENCFF042RAK	KDM4B	8167	1268	15.53
ENCFF714XHL	BRD4	8192	1262	15.41
ENCFF557DSM	YY1	5213	803	15.40
ENCFF722XRW	SRSF3	1984	304	15.32
ENCFF641LJY	THAP1	5972	914	15.30
ENCFF778RMG	GTF2E2	16371	2500	15.27
ENCFF990CFV	POU5F1	2219	331	14.92
ENCFF059WVE	SRSF7	208	31	14.90
ENCFF122QSN	HNRNPLL	15382	2261	14.70
ENCFF058NMZ	SP1	14133	2074	14.67
ENCFF117EWO	CSDE1	5866	857	14.61
ENCFF191MPD	SIN3A	13459	1950	14.49

Datasets	Genes	Total Peaks	Overlap Peaks	Overlap Percentages (%)
ENCFF682SIY	RBM14	3099	448	14.46
ENCFF280GTU	SIN3B	10501	1518	14.46
ENCFF941RVL	PCBP1	10920	1541	14.11
ENCFF626KTJ	PHF8	29046	3972	13.67
ENCFF315YAF	SMAD1	8855	1196	13.51
ENCFF253FON	ZBED1	5913	779	13.17
ENCFF048OBR	TBL1XR1	6100	799	13.10
ENCFF178MOP	SMAD5	22325	2924	13.10
ENCFF030HWZ	ATF1	3724	485	13.02
ENCFF379MPS	RBBP5	24338	3127	12.85
ENCFF718NUA	SAP30	14058	1799	12.80
ENCFF368TYM	KDM5B	22700	2904	12.79
ENCFF998YJY	E2F1	9347	1193	12.76
ENCFF010STZ	HNRNPL	8012	1018	12.71
ENCFF801YZV	POLR2H	23424	2956	12.62
ENCFF998HOR	HDAC6	1761	222	12.61
ENCFF602CFZ	ZNF407	5119	632	12.35
ENCFF470MLY	MBD2	15555	1910	12.28
ENCFF209JJD	RBM39	2747	337	12.27
ENCFF938IOJ	UBTF	9567	1165	12.18
ENCFF434QZL	ZBTB11	7250	873	12.04
ENCFF785HXY	TAF9B	15392	1853	12.04
ENCFF474NLG	HCFC1	13559	1621	11.96
ENCFF701MXF	TFDP1	22661	2709	11.95
ENCFF484DKT	CBX1	14582	1722	11.81
ENCFF303PMJ	ZSCAN29	5484	645	11.76
ENCFF249EZR	TCF7	5844	685	11.72
ENCFF478MPX	SAFB	5923	684	11.55
ENCFF380FJL	TBP	21570	2489	11.54
ENCFF937VZI	POLR2A	25816	2954	11.44
ENCFF415YPX	ILF3	4762	541	11.36
ENCFF594LCH	NR2C2	634	72	11.36
ENCFF582YPB	CREB3L1	22194	2519	11.35
ENCFF872JJJ	POLR2B	20684	2333	11.28
ENCFF970LCB	MXI1	9033	1016	11.25
ENCFF588OLK	ZZZ3	3029	340	11.22
ENCFF522JRK	ZC3H11A	2624	293	11.17
ENCFF948YVM	PHF20	15696	1742	11.10
ENCFF624NUZ	SAFB2	82	9	10.98
ENCFF925DJG	E2F8	12738	1397	10.97

Datasets	Genes	Total Peaks	Overlap Peaks	Overlap Percentages (%)
ENCFF120IDE	RBFOX2	56370	6120	10.86
ENCFF433RKB	ZFX	27044	2923	10.81
ENCFF617CAZ	TAF15	787	85	10.80
ENCFF484HCG	SUPT5H	29659	3187	10.75
ENCFF881QBT	PML	21322	2271	10.65
ENCFF310AGG	E2F7	3169	333	10.51
ENCFF717TWA	KLF1	11788	1234	10.47
ENCFF357NRL	HDAC1	17258	1801	10.44
ENCFF973OME	MNT	22571	2339	10.36
ENCFF526EEI	ELF1	20422	2110	10.33
ENCFF235ZZP	ASH1L	8152	839	10.29
ENCFF120WOF	ZNF639	26368	2700	10.24
ENCFF475TQE	ATF3	7705	772	10.02
ENCFF685KAG	GABPA	33376	3342	10.01
ENCFF422BXE	RUNX1	3657	366	10.01
ENCFF011KRN	SMAD2	200	20	10.00
ENCFF289BUU	NUFIP1	3572	356	9.97
ENCFF925ANU	EP400	33534	3328	9.92
ENCFF898FAQ	ZNF740	8099	792	9.78
ENCFF581YKY	NEUROD1	14353	1399	9.75
ENCFF802XZN	KLF13	11910	1158	9.72
ENCFF312RFN	TCF7L2	2119	206	9.72
ENCFF581ZZT	ZNF282	12961	1253	9.67
ENCFF033EBX	SIX5	3674	350	9.53
ENCFF209HKW	RLF	10587	998	9.43
ENCFF384YDT	IRF2	8509	802	9.43
ENCFF768TJI	HNRNPUL1	1122	105	9.36
ENCFF972LPT	RNF2	17627	1647	9.34
ENCFF906PGD	NKRF	18069	1686	9.33
ENCFF338HQY	DDX20	10079	940	9.33
ENCFF481WGW	SKIL	24313	2257	9.28
ENCFF144PPR	NRF1	21560	1994	9.25
ENCFF296CNQ	POLR2AphosphoS2	10069	928	9.22
ENCFF294EHN	SETDB1	4113	375	9.12
ENCFF211XFR	ZC3H8	3583	325	9.07
ENCFF777CWV	HNRNPK	11589	1047	9.03
ENCFF998KKJ	XRCC5	13253	1196	9.02
ENCFF350YXB	MTA3	28818	2581	8.96
ENCFF126BCK	GMEB1	27813	2479	8.91
ENCFF583GKE	CCAR2	543	48	8.84

Datasets	Genes	Total Peaks	Overlap Peaks	Overlap Percentages (%)
ENCFF408SNO	DIDO1	7905	695	8.79
ENCFF359QCN	ZBTB5	4857	427	8.79
ENCFF522JUV	RBM22	13050	1137	8.71
ENCFF167HYX	GTF2A2	6423	553	8.61
ENCFF836VRV	AGO1	11428	983	8.60
ENCFF043YZF	LEF1	5184	445	8.58
ENCFF517KRT	GABPB1	30655	2622	8.55
ENCFF322DSX	NCOA6	4645	392	8.44
ENCFF545VNY	ETV6	6732	567	8.42
ENCFF502KHR	ELF4	21065	1773	8.42
ENCFF494ZUY	SNIP1	3268	272	8.32
ENCFF273EYJ	POLR2G	58571	4874	8.32
ENCFF975IRM	RB1	25768	2129	8.26
ENCFF344QKL	ZNF274	3064	252	8.22
ENCFF542DOG	POLR2AphosphoS5	30324	2485	8.19
ENCFF134CUM	PHF21A	8976	732	8.16
ENCFF966MVC	SUZ12	2808	227	8.08
ENCFF694BIA	PTBP1	5027	400	7.96
ENCFF356ASJ	E2F5	19472	1544	7.93
ENCFF643TEZ	CDC5L	5690	448	7.87
ENCFF186QUP	VEZF1	46383	3646	7.86
ENCFF772HOY	ETS2	1762	138	7.83
ENCFF388YOB	ZNF395	25387	1980	7.80
ENCFF197OGH	MLLT1	13522	1047	7.74
ENCFF723RSX	NFATC3	13570	1047	7.72
ENCFF549KOD	GATAD2B	13320	1019	7.65
ENCFF772QPO	KAT8	2268	173	7.63
ENCFF597DIY	ZMIZ1	1049	80	7.63
ENCFF853TZC	ZNF197	10450	795	7.61
ENCFF654BMD	ZNF83	11272	856	7.59
ENCFF465JKF	MYC	31436	2356	7.49
ENCFF616LVN	MITF	7602	569	7.48
ENCFF507MGL	ARNT	11519	861	7.47
ENCFF074EZL	NBN	20957	1566	7.47
ENCFF076MSV	AFF1	9959	743	7.46
ENCFF083YCQ	E4F1	28697	2136	7.44
ENCFF262BVP	TOE1	19775	1461	7.39
ENCFF561USY	ZBTB2	18423	1355	7.35
ENCFF706ISJ	ZBTB7A	30583	2209	7.22
ENCFF939ZFS	HDAC8	9908	714	7.21

Datasets	Genes	Total Peaks	Overlap Peaks	Overlap Percentages (%)
ENCFF116DIO	ZNF148	22956	1646	7.17
ENCFF396NSO	HLTF	12623	885	7.01
ENCFF996IVU	ZNF830	3460	242	6.99
ENCFF246HIU	YBX3	2680	187	6.98
ENCFF163VUK	ZKSCAN1	14153	987	6.97
ENCFF917COW	E2F6	32326	2193	6.78
ENCFF881XQF	PCBP2	2539	172	6.77
ENCFF452SPE	FOXJ2	15441	1042	6.75
ENCFF004WYV	EGR1	30502	2023	6.63
ENCFF417LEJ	ZBTB40	29759	1964	6.60
ENCFF492GXZ	FOXK2	23741	1561	6.58
ENCFF666LZV	BRD9	18855	1226	6.50
ENCFF034PQD	DNMT1	3604	234	6.49
ENCFF511OXK	LARP7	9680	624	6.45
ENCFF868QLL	ATF7	47113	3035	6.44
ENCFF657VJX	HDAC2	5292	339	6.41
ENCFF173NRW	ZNF644	12705	805	6.34
ENCFF835YLR	ID3	24733	1561	6.31
ENCFF858QMI	TRIM28	14198	857	6.04
ENCFF253FZN	GATAD2A	15131	905	5.98
ENCFF602OKQ	ESRRA	33326	1989	5.97
ENCFF314ULQ	L3MBTL2	53984	3204	5.94
ENCFF238MYM	ZNF24	18184	1072	5.90
ENCFF080NBZ	NCOR1	8768	513	5.85
ENCFF602AXP	ZNF589	21343	1243	5.82
ENCFF769GYG	MTA1	17990	1045	5.81
ENCFF799HIG	MAX	38228	2219	5.80
ENCFF152VMJ	CTBP1	48724	2756	5.66
ENCFF962VSZ	KHSRP	5187	293	5.65
ENCFF421KIV	PYGO2	4223	238	5.64
ENCFF662MVX	MTA2	20748	1161	5.60
ENCFF365NKO	MIER1	9498	530	5.58
ENCFF574LAO	TRIM25	5616	311	5.54
ENCFF948TXN	CREM	38796	2121	5.47
ENCFF877ANE	MYBL2	4580	247	5.39
ENCFF583DZD	MYNN	15422	825	5.35
ENCFF396DNK	ZFP91	14258	762	5.34
ENCFF404YMX	ETV1	24198	1273	5.26
ENCFF455TDM	CBX3	26746	1406	5.26
ENCFF248AOD	ZNF766	26482	1386	5.23

Datasets	Genes	Total Peaks	Overlap Peaks	Overlap Percentages (%)
ENCFF417TXD	USF2	17333	899	5.19
ENCFF676NPW	HES1	11091	572	5.16
ENCFF269EMM	PBX2	3922	202	5.15
ENCFF010MMQ	ARHGAP35	5027	258	5.13
ENCFF484CKD	RCOR1	6822	349	5.12
ENCFF033KXY	SNRNP70	882	45	5.10
ENCFF664ZGR	NR2C1	15500	788	5.08
ENCFF815TTY	ZNF584	15035	745	4.96
ENCFF721LDD	ZNF175	18917	933	4.93
ENCFF859NPS	NR4A1	20688	1013	4.90
ENCFF241TBP	SOX6	37311	1810	4.85
ENCFF062ARS	CBX5	7083	342	4.83
ENCFF179NDS	BHLHE40	27327	1317	4.82
ENCFF558VPP	REST	14541	700	4.81
ENCFF922TDM	NCOA2	3903	187	4.79
ENCFF629BFI	JUN	21668	1036	4.78
ENCFF561WNI	HDGF	11445	542	4.74
ENCFF067LIW	MCM2	423	20	4.73
ENCFF197YHU	SMARCA4	31603	1494	4.73
ENCFF426DUB	JUNB	5347	250	4.68
ENCFF048OIZ	BMI1	2818	131	4.65
ENCFF699ZII	FOXM1	18535	855	4.61
ENCFF670ZCR	MGA	33894	1560	4.60
ENCFF452SVO	MCM7	2800	128	4.57
ENCFF580DYE	HDAC3	10781	474	4.40
ENCFF294HEI	PRDM10	29758	1295	4.35
ENCFF921WMN	ZNF318	10963	473	4.31
ENCFF495CSO	NCOA4	3079	132	4.29
ENCFF695CPH	CUX1	6966	294	4.22
ENCFF859FVX	CHAMP1	14318	598	4.18
ENCFF561IZB	TRIP13	2520	105	4.17
ENCFF556YCY	ZNF184	10515	438	4.17
ENCFF549TYR	EP300	28288	1152	4.07
ENCFF835KAT	ARID3A	11550	470	4.07
ENCFF423EMU	BACH1	4708	190	4.04
ENCFF273TYA	ZNF592	35259	1420	4.03
ENCFF255EOB	NR2F2	21050	828	3.93
ENCFF407AOX	ZEB2	23292	913	3.92
ENCFF294VWT	THRAP3	4904	191	3.89
ENCFF096XMD	RAD51	17974	675	3.76

Datasets	Genes	Total Peaks	Overlap Peaks	Overlap Percentages (%)
ENCFF580QGA	RELA	10319	385	3.73
ENCFF696KPD	STAT5A	13378	494	3.69
ENCFF337DKJ	JUND	47775	1676	3.51
ENCFF209MQX	TEAD4	35771	1252	3.50
ENCFF646VQW	TRIM24	34485	1181	3.42
ENCFF874QUM	CC2D1A	26447	902	3.41
ENCFF944GJH	SMARCB1	3079	105	3.41
ENCFF529FWJ	ZKSCAN8	11311	381	3.37
ENCFF257RYT	MEF2D	14762	493	3.34
ENCFF265LKN	HINFP	8293	272	3.28
ENCFF114IWY	ZNF143	29873	958	3.21
ENCFF692PVV	FOSL1	58938	1887	3.20
ENCFF577UJR	MEF2A	1348	43	3.19
ENCFF370ENX	NFIC	48576	1538	3.17
ENCFF462FRU	PHB2	7367	233	3.16
ENCFF706SJZ	ZNF384	19825	626	3.16
ENCFF665FHC	CREB3	14139	446	3.15
ENCFF774QKS	DPF2	26101	797	3.05
ENCFF622RBW	SMARCE1	36356	1103	3.03
ENCFF175IIE	NR2F1	37884	1074	2.83
ENCFF544XKC	PKNOX1	52588	1451	2.76
ENCFF526HLP	DACH1	19460	535	2.75
ENCFF886VSU	IKZF1	49278	1353	2.75
ENCFF820EVZ	EWSR1	1795	49	2.73
ENCFF114PTZ	SMARCC2	19106	513	2.69
ENCFF242IWJ	TCF12	16250	434	2.67
ENCFF057ZUY	BCOR	44533	1154	2.59
ENCFF396GUH	ADNP	21217	538	2.54
ENCFF613RNG	MEIS2	53002	1338	2.52
ENCFF684MLP	COPS2	123	3	2.44
ENCFF521CRG	ZFP36	23635	574	2.43
ENCFF995NIE	SMARCA5	19278	463	2.40
ENCFF712AXK	NR2F6	23333	551	2.36
ENCFF844HBQ	KLF16	17908	415	2.32
ENCFF744SVC	ZNF354B	4779	108	2.26
ENCFF986MHU	NFXL1	6160	135	2.19
ENCFF237UAN	RFX1	18387	402	2.19
ENCFF482CEV	TAL1	28775	629	2.19
ENCFF196DHR	IRF9	9536	205	2.15
ENCFF332ICQ	ARID2	11835	254	2.15

Datasets	Genes	Total Peaks	Overlap Peaks	Overlap Percentages (%)
ENCFF388AJH	IRF1	17589	370	2.10
ENCFF996ZGL	C11orf30	61206	1282	2.09
ENCFF619BDC	TEAD2	6623	135	2.04
ENCFF664XPS	SPI1	32255	648	2.01
ENCFF558DSF	HMBOX1	28923	581	2.01
ENCFF632NQI	GATA1	14605	291	1.99
ENCFF225MPC	ARID1B	50204	932	1.86
ENCFF680WBN	RBM25	49721	875	1.76
ENCFF806BDC	EHMT2	19437	342	1.76
ENCFF041YQC	SMC3	26872	472	1.76
ENCFF495MHZ	NFE2	30144	517	1.72
ENCFF710IEF	ATF4	42275	715	1.69
ENCFF176NOI	ILK	2284	37	1.62
ENCFF732HOE	GATA2	11622	186	1.60
ENCFF803LEC	XRCC3	1986	29	1.46
ENCFF738WCE	KDM1A	46025	672	1.46
ENCFF812QPN	MAFK	26965	390	1.45
ENCFF442ZUF	ZNF316	19004	246	1.29
ENCFF085HTY	CTCF	48729	617	1.27
ENCFF993GXU	CBFA2T3	53515	670	1.25
ENCFF968KBN	ATF2	46506	560	1.20
ENCFF642BNC	CBFA2T2	32296	386	1.20
ENCFF429XKT	CEBPB	46348	529	1.14
ENCFF671BFH	THRA	1757	19	1.08
ENCFF606CCB	CEBPG	37381	389	1.04
ENCFF308IXJ	MAFF	27637	283	1.02
ENCFF007DKB	PTRF	5154	43	0.83
ENCFF332DRG	PTTG1	5547	46	0.83
ENCFF809YFY	FOXA1	1350	11	0.81
ENCFF954NAJ	NR0B1	405	3	0.74
ENCFF398EQF	U2AF2	137	1	0.73
ENCFF872YNU	MCM5	431	3	0.70
ENCFF447QUG	ZNF512	23246	151	0.65
ENCFF549GMO	BRCA1	951	6	0.63
ENCFF706LIT	YBX1	1358	8	0.59
ENCFF511QHY	ZMYM3	39230	205	0.52
ENCFF927JBT	MAFG	46186	217	0.47
ENCFF175IUO	NFE2L1	7430	31	0.42
ENCFF386WKY	ZNF280A	6438	24	0.37
ENCFF408FQC	ZBTB33	60931	218	0.36

Datasets	Genes	Total Peaks	Overlap Peaks	Overlap Percentages (%)
ENCFF679ITJ	MCM3	2788	9	0.32
ENCFF036YVI	TSC22D4	3352	9	0.27
ENCFF834WGJ	ZNF507	11748	11	0.09
ENCFF137IBM	SRSF9	29	0	0.00

Table S3. A list of DNA and RNA probes for *in vitro* assays.

Probes	Sequences
cMYC_G4	5′-TAMRA-TGAGGGTGGGGAGGGTGGGGAAGG -3′
cMYC_M4	5′-TAMRA-TGAGGGTGA GGAGTG TGGGGAAGG-3′
cKIT_G4	5′-TAMRA-AGGGAGGGCGCTGGGAGGAGG G-3′
cKIT M4	5′-TAMRA-AGGGAGGGCTCTGTGAGGAGG G-3′
hTel_G4	5′-TAMRA-AAAGGGTTAGGGTTAGGGTTAGGGAA-3′
hTel_M4	5′-TAMRA-AAAGGGTTAGTGTTAGTGTTAGGGAA-3′
2KF8_G4	5′-TAMRA-GGGTTAGGGTTAGGGTTAGGGT-3′
2KF8_M4	5′-TAMRA-GGGTTAGTGTTAGTGTTAGGGT-3′
U2AF1_RNA Motif	5'-Cy3-UUUUAGGU-3'
RNA/DNA_RNA strand	5′-Cy3-GCGAGGGGCGGCCGGAGAGC-3'
RNA/DNA_DNA strand	5′-GCTCTCCGGCCGCCCCTCGC-3'
R-loop_RNA strand	5'-Cy3-GCGAGGGGCGGCCGGAGAGC-3'
R-loop_Templated DNA strand	5'- CCGCTCCGGCAGCTCAGGCCCTGGCGCTCTCCGGCCGCCCC TCGCCCGCCCGGGCTCCTCCTCCTGTGCC-3'
cMYC-R-loop_G4 Non-templated DNA strand	5'- GGCACAGGAGGAGGAGTTTTTTCTGAGGGTGGGGAGGGTGG GGAAGGCATTTTTTGAGCTGCCGGAGCGG-3'
cMYC-R-loop_M4 Non-templated DNA strand	5'- GGCACAGGAGGAGGAGTTTTTTCTGAGGGTGAGGAGTGTGG GGAAGGCATTTTTTGAGCTGCCGGAGCGG-3'
dsDNA_Non-templated strand	5′-Cy3-GCGAGGGGCGGCCGGAGAGC-3'
dsDNA_Templated strand	5′-GCTCTCCGGCCGCCCCTCGC-3'
cKIT-G4-R-loop_ Non-templated DNA strand	GGCACAGGAGGAGGAGTTTTTTCAGGGAGGGCGCTGGGAGG AGGGCATTTTTTGAGCTGCCGGAGCGG
cKIT-M4-R-loop_ Non-templated DNA strand	GGCACAGGAGGAGGAGTTTTTTCAGGGAGGGCTCTGTGAGG AGGGCATTTTTTGAGCTGCCGGAGCGG

Table S4. A list of shRNA sequences for the stable knockdown experiments.

**Names**	**Sequences**
shCtrl	TTACGTGACACGTTCGGAGAA
shU2AF1-1	ATCATAGTGTTCCTGCATCTC
shU2AF1-2	AATGGTCTGGCTAAACGTCGG
shDHX9-1	TATATCCGCTTCCATTGTCGT
shDHX9-2	GCATAAAACTTCTGCGTCT

Table S5. A list of primers for RT-PCR experiments.

Gene Names	Forward primers	Reverse primers
SRRM1	AGACATTCCCCTTCCCGGAGT	GAAGGAGACCGTCGCCTTCT
SETX	TCACACGAGCCAAGTACAGC	GCACAGGCTTGAGTTTCAGA
EIF4A2	GCTCGCGGGATTGATGTGC	GAATCCTCTTGTCTTCTTC
CHEK2	CAGCTCTCAATGTTGAAACAGAA	CTGCACAGCCAAGAGCATC
LENG8	CAGCAGTACAACTATGCCTACC	CAGCTGTTGGCCCTTCTT
GSK3B	GAACTCCAACAAGGGAGCAA	GTGGTGTTAGTCGGGCAGTT

Table S6. A list of primers for ChIP-qPCR experiments.

Gene Names	Forward primes	Reverse primers
SRRM1	CCGAAACCGAAACCCATCTA	CCATGAAGCGATTGAAGAGAAAG
SETX	CTCAGGTGTCTCAGCGGATG	CGCATTGTTCGCAAGACCTA
EIF4A2	GTGGGAGCAAGCGGAATAA	CTCGCGGCGGAAGAATATAG
CHEK2	GAGAGCGTCTAACCAGACTAATG	ACCACCAAACACCCAACA
GSK3B	CTTCACCAATCACCGAAGGA	AAGGTAGCCGAACGGAAAG
LENG8	CGCACTTACGCATGAACATT	AGACTCCGTCTCCGAGAACA

Table S7. A list of primers for CLIP-qPCR experiments.

Gene Names	Forward primes	Reverse primers
SRRM1	ATGGGAAAGCGATGGCAA	AGCTCATGTATAATTCTCCC
SETX	CAACTTCTTCCTAGGAGCTT	CTTGAGAGCCCAGTGTAGAT
CHEK2	AGGAATGAACCCCTTGCCTT	CTGCACAGCCAAGAGCATC
LENG8	ATGAGAGCATGTCCTACCAG	CAGCTGTTGGCCCTTCTT

Figure S1. Gene annotation results revealing that U2AF1 is predominantly enriched at promoter regions in human cells. Gene annotations of U2AF1 ChIP-seq peaks (A), overlapped peaks of U2AF1 ChIP-seq and BG4-ChIP-seq datasets (B), overlapped peaks of U2AF1 ChIP-seq and R-ChIP-seq datasets (C), and overlapped peaks among U2AF1 ChIP-seq, BG4-ChIP-seq and R-ChIP-seq datasets (D).

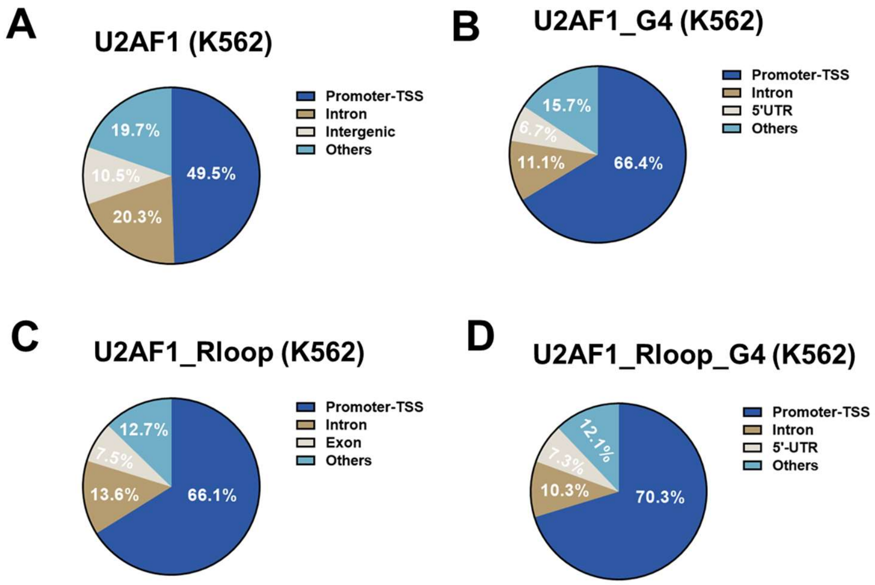

Figure S2. The binding preferences of U2AF1 protein are recapitulated in HepG2 cells. (A-B) Overlap analysis of U2AF1 ChIP-seq and BG4-ChIP-seq datasets. Gene annotations of U2AF1 ChIP-seq peaks (C), and overlapped peaks of U2AF1 and BG4-ChIP-seq datasets (D).

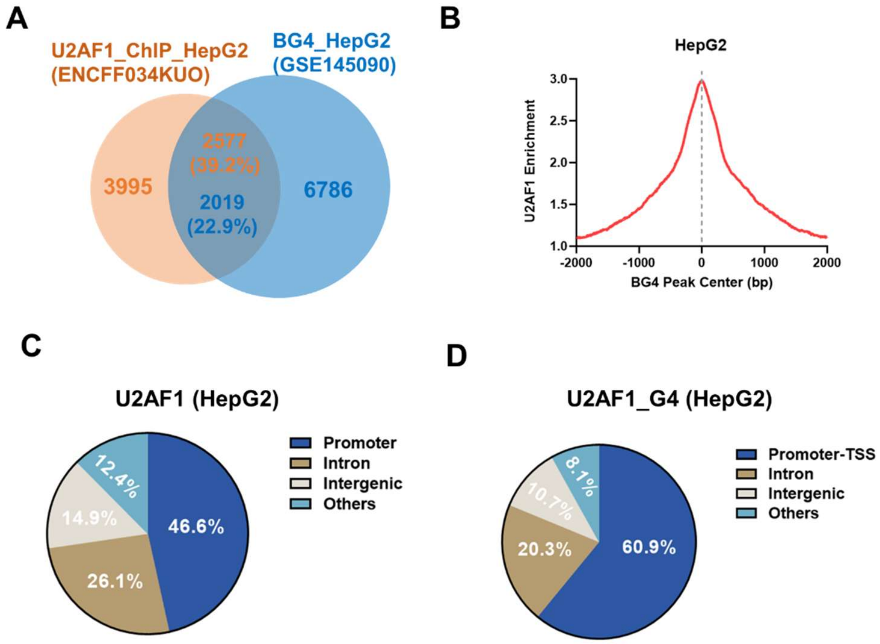

Figure S3. Fluorescence anisotropy for examining the binding of full-length U2AF1 and its zinc finger domains toward G4, M4 and dsDNA probes. (A) SDS-PAGE images showing the purities of recombinant full-length His-U2AF1 protein, His-U2AF1-ZF1 and His-U2AF1-ZF2 proteins. (B-D) Fluorescence anisotropy results showing the binding affinities of U2AF1 toward G4 and M4 probes derived from *cKIT* (B) and human telomeric repetitive sequences 2KF8 (C) and hTel (D). Error bars represent S.E.M. (n = 3). (E) Native PAGE gel showing the successful formation of R-loop structures. (F) Fluorescence anisotropy results showing the binding affinities of U2AF1 toward dsDNA.

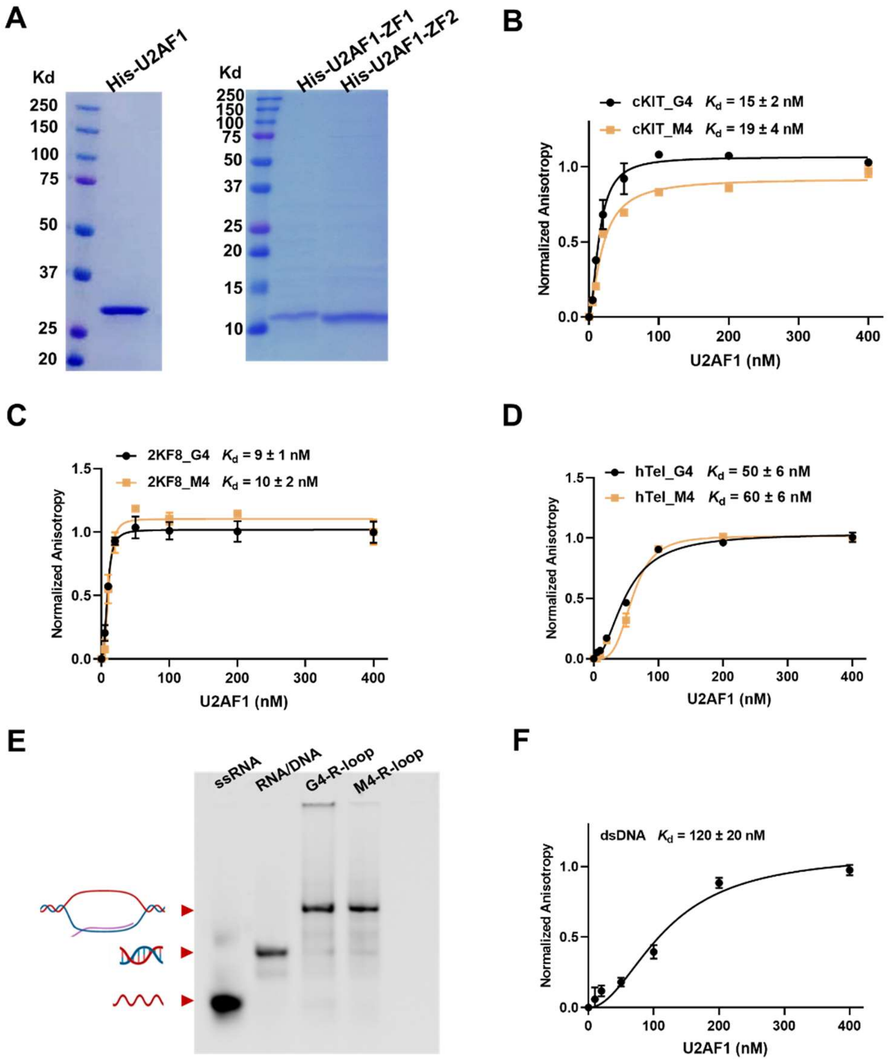

Figure S4. EMSA results for full-length U2AF1 protein toward G4 and M4 probes.

EMSA gel images and binding curves of U2AF1 toward cMYC_G4 (A), cMYC_M4 (B), cKIT_G4 (C), cKIT_M4 (D) in K^+ buffer, and cMYC_G4 in Li^+ buffer (E). Error bars represent S.E.M. (n = 3).

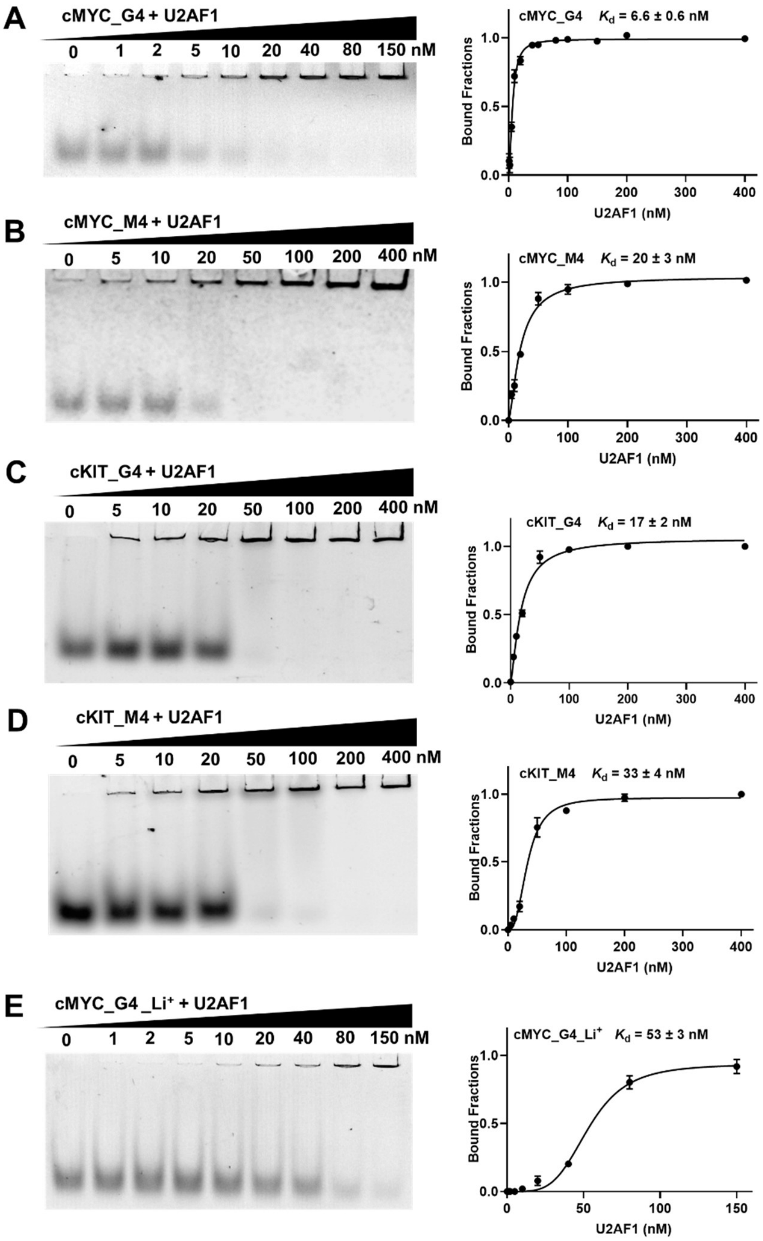

Figure S5. Circular dichroism (CD) spectroscopy confirms the successful formation of G4 and R-loop structures under our experimental conditions. (A) CD spectra of cMYC G4 probe (5 μM) with or without U2AF1 protein, as well as U2AF1 alone. (B) CD spectrum of G4-R-loop and M4-R-loop probes (5 μM each).

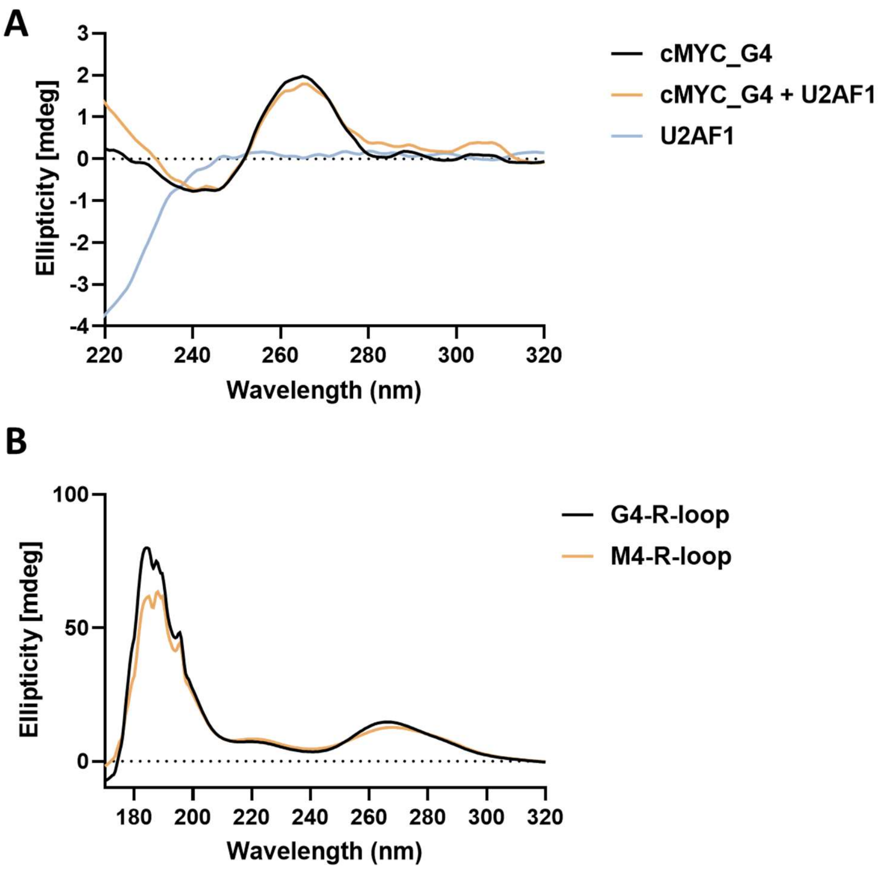

Figure S6. EMSA results for full-length U2AF1 protein toward R-loop probes. EMSA gel images and binding curves of U2AF1 toward G4-R-loop (A), and M4-R-loop probes (B). Error bars represent S.E.M. (n = 3). Red arrow in (A) indicates the formation of putative intermolecular G4 structure in G4-R-loop.

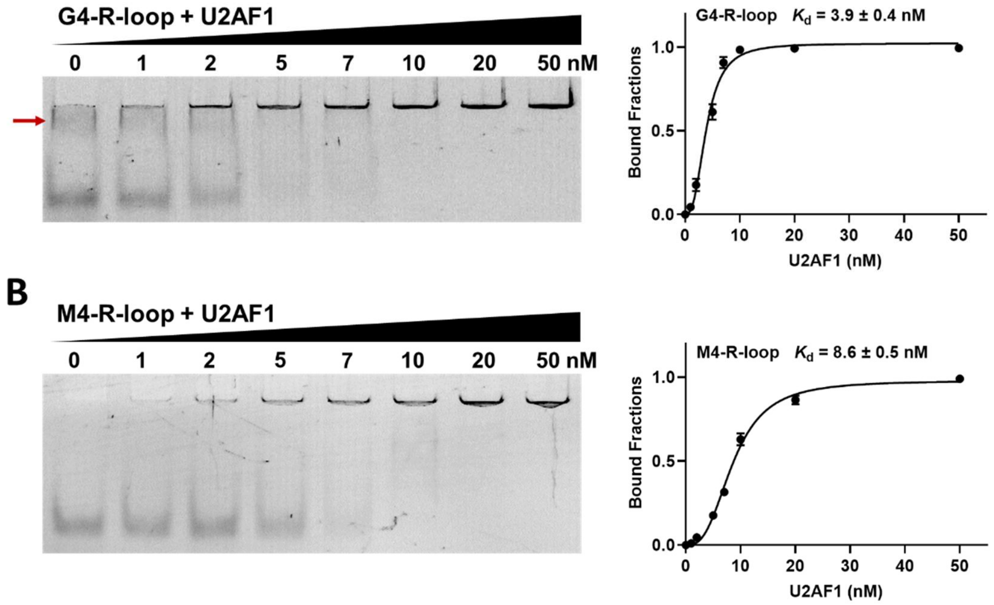

Figure S7. U2AF1-Rloop interaction elicits phase separation of U2AF1 protein *in vitro*. (A) Fluorescence anisotropy result showing the abnormal anisotropy values of Cy3-Rloop when incubated with high concentrations of U2AF1 protein. (B) Fluorescence images showing that U2AF1-cMYC_R-loop mixture undergoes phase separation when the ratios of Cy3-Roop to U2AF1 being above 1: 5. The concentrations of Cy3-Rloop in these solutions are fixed at 100 nM. The ratios of Cy3-Rloop to U2AF1 are annotated in the upper left corner of each image. (C) Fluorescence images showing that U2AF1-cKIT_R-loop mixture undergoes phase separation.

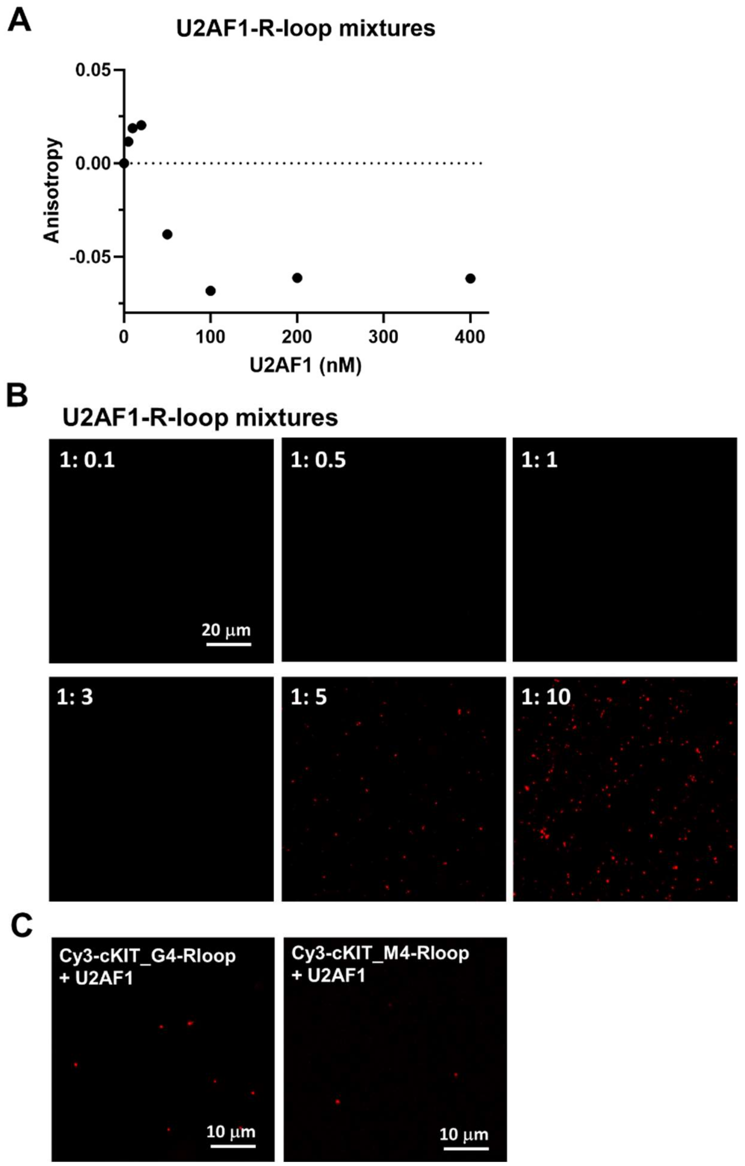

Figure S8. U2AF1 undergoes phase separation, which could be enhanced by dextran and R-loop, while disrupted by 1,6-HD. (A-C) DIC images of U2AF1 (A), U2AF1 with 10% dextran (B), and U2AF1 with 10% dextran and 5% 1,6-HD (C). The concentrations of U2AF1 in these solutions are 500 nM. (D) Bar plots showing the comparison of droplets number in different conditions. Green solid triangles indicate the locations of droplets.

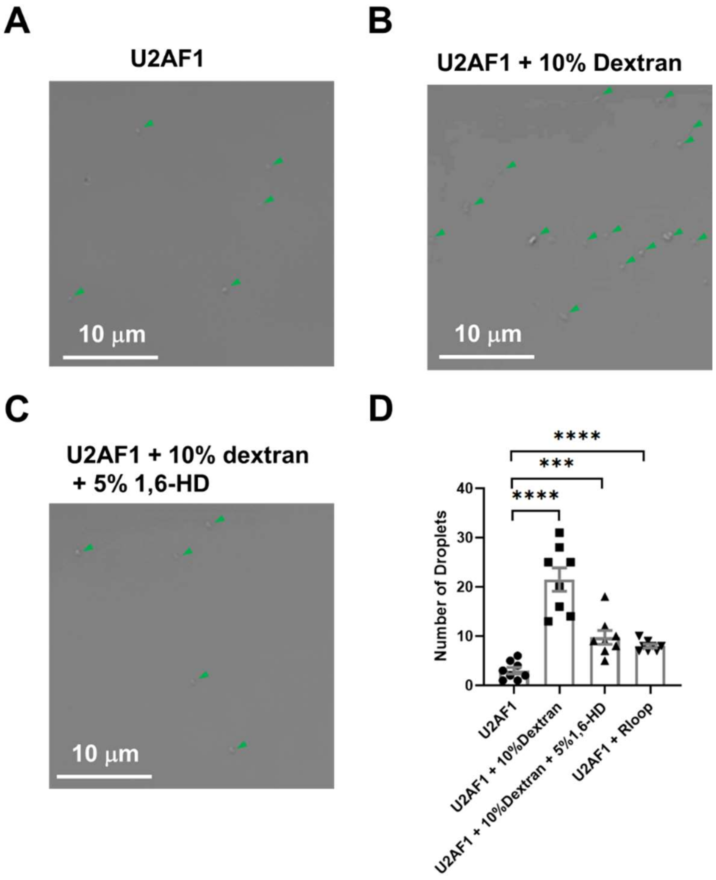

Figure S9. The material properties of U2AF1 droplets are similar to those of prion-like RNA binding proteins. (A) SDS-PAGE gel image of recombinant EGFP-U2AF1 protein. (B) Fluorescence images of Cy3-Rloop-U2AF1 mixtures with increasing Cy3-Rloop/U2AF1 ratios, which were shown in upper left corner of each image. The experiments were performed with 1 μM of unlabeled U2AF1 proteins. (C) IUPred2 prediction indicating RS domain of U2AF1 is an intrinsically disordered region. A score of more than 0.5 indicates disordered.

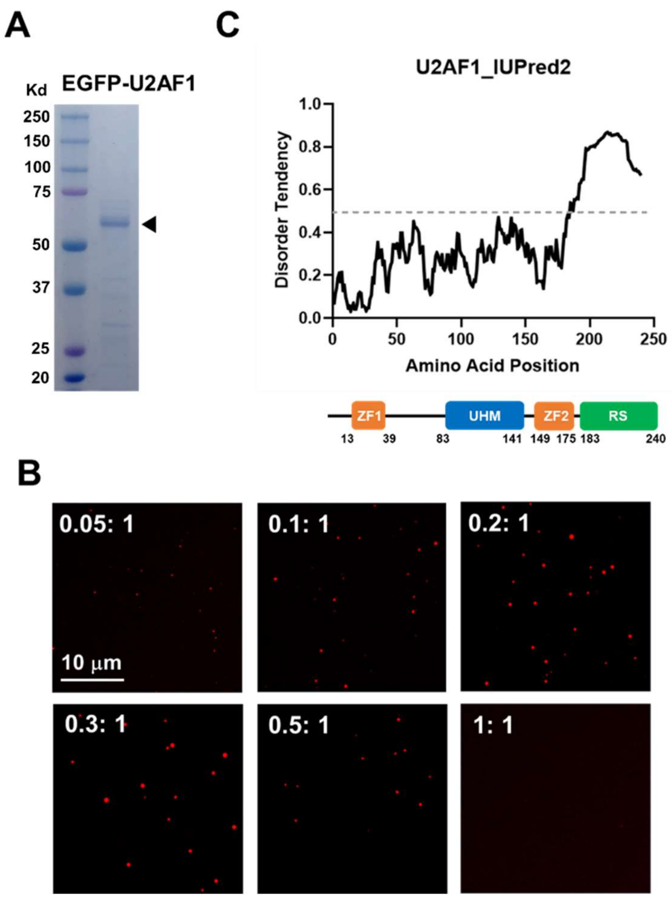

Figure S10. The ZF domains contribute to the phase separation property of U2AF1. (A-B) The removal of either ZF1 (A) or ZF2 (B) domain results in a diminished foci formation of U2AF1 in cells. (C) Live-cell imaging showing that U2AF1 undergoes phase separation in cells, which can be disrupted by 1,6-HD.

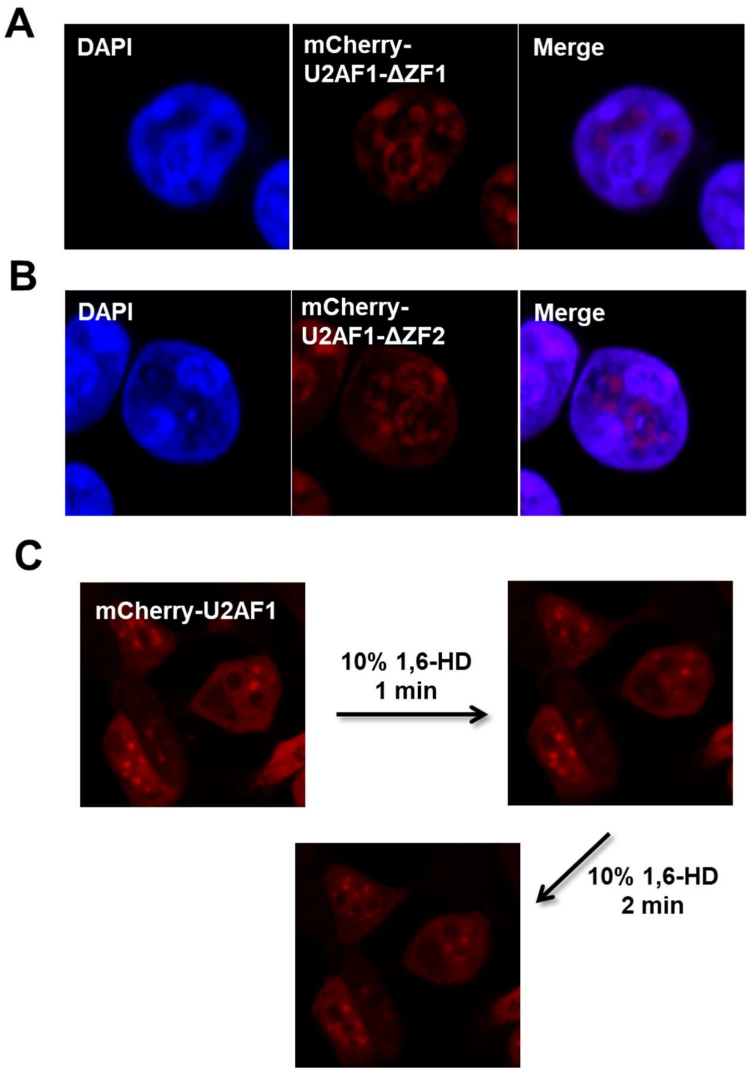

Figure S11. R-ChIP-qPCR and RNAPII-ChIP-seq results showing the occupancy of R-loops and RNAPII in promoters, respectively. (A) R-ChIP-qPCR for the promoters of *SRRM1*, *SETX*, *EIF4A2*, *LENG8*, and *CHEK2* genes. Error bars represent S.E.M. (n = 3). The p values were calculated by using unpaired, two-tailed Student's t-test. *, $0.01 \le p < 0.05$; **, $0.001 \le p < 0.01$; ***, $p < 0.001$. (B) RNAPII-ChIP-qPCR results showing decreased and increased occupancies of RNAPII in promoter regions of the indicated genes upon genetic depletions of U2AF1 and DHX9 depletion, respectively. (C) IGV plots showing the RNAPII enrichment in promoter of *LENG8* gene in these cell lines.

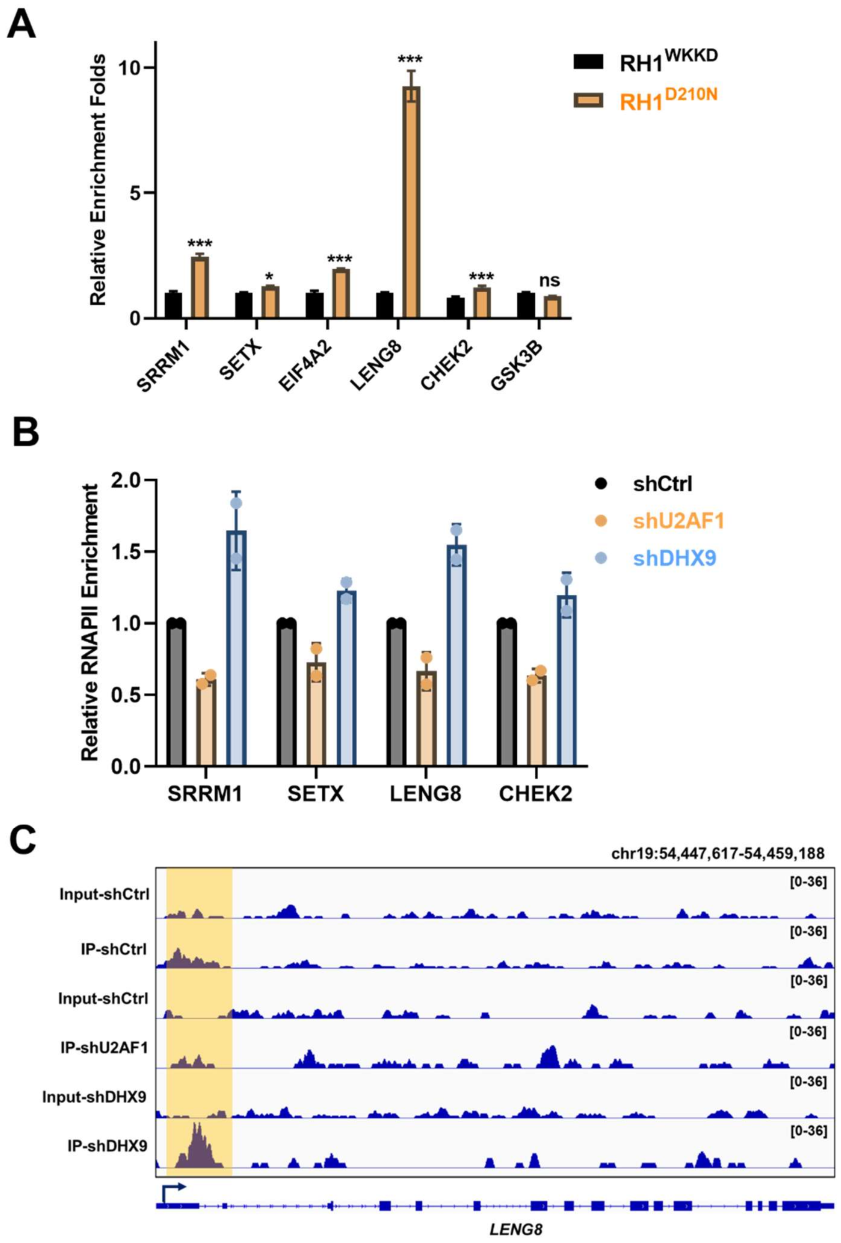

Supplement: Supplementary file 1 — ja3c08204_si_001.pdf [file ja3c08204_si_001.pdf]
